# A fungal transcription factor essential for starch degradation affects integration of carbon and nitrogen metabolism

**DOI:** 10.1371/journal.pgen.1006737

**Published:** 2017-05-03

**Authors:** Yi Xiong, Vincent W. Wu, Andrea Lubbe, Lina Qin, Siwen Deng, Megan Kennedy, Diane Bauer, Vasanth R. Singan, Kerrie Barry, Trent R. Northen, Igor V. Grigoriev, N. Louise Glass

**Affiliations:** 1 The Department of Plant and Microbial Biology, The University of California, Berkeley, California, United States of America; 2 The Energy Biosciences Institute, The University of California, Berkeley, California, United States of America; 3 Environmental Genomics and System Biology/Biosciences Area Lawrence Berkeley National Laboratory, Berkeley, California, United States of America; 4 U.S. Department of Energy Joint Genome Institute, Walnut Creek, California, United States of America; 5 Joint BioEnergy Institute, Emeryville, California, United States of America; HudsonAlpha Institute for Biotechnology, UNITED STATES

## Abstract

In *Neurospora crassa*, the transcription factor COL-26 functions as a regulator of glucose signaling and metabolism. Its loss leads to resistance to carbon catabolite repression. Here, we report that COL-26 is necessary for the expression of amylolytic genes in *N*. *crassa* and is required for the utilization of maltose and starch. Additionally, the Δ*col-26* mutant shows growth defects on preferred carbon sources, such as glucose, an effect that was alleviated if glutamine replaced ammonium as the primary nitrogen source. This rescue did not occur when maltose was used as a sole carbon source. Transcriptome and metabolic analyses of the Δ*col-26* mutant relative to its wild type parental strain revealed that amino acid and nitrogen metabolism, the TCA cycle and GABA shunt were adversely affected. Phylogenetic analysis showed a single *col-26* homolog in Sordariales, Ophilostomatales, and the Magnaporthales, but an expanded number of *col-26* homologs in other filamentous fungal species. Deletion of the closest homolog of *col-26* in *Trichoderma reesei*, *bglR*, resulted in a mutant with similar preferred carbon source growth deficiency, and which was alleviated if glutamine was the sole nitrogen source, suggesting conservation of COL-26 and BglR function. Our finding provides novel insight into the role of COL-26 for utilization of starch and in integrating carbon and nitrogen metabolism for balanced metabolic activities for optimal carbon and nitrogen distribution.

## Author summary

In nature, filamentous fungi sense nutrient availability in the surrounding environment and adjust their metabolism for optimal utilization, growth and reproduction. Carbon and nitrogen are two of major elements required for life. Within cells, signals from carbon and nitrogen catabolism are integrated, resulting in balanced metabolic activities for optimal carbon and nitrogen distribution. However, coordination of carbon and nitrogen metabolism is often missed in studies that are based on comparisons between single carbon or nitrogen sources. In this study, we performed systematic transcriptional profiling of *Neurospora crassa* on different components of starch and identified the transcription factor COL-26 to be an essential regulator for starch utilization and needed for coordinating carbon and nitrogen regulation and metabolism. Proteins with sequence similar to COL-26 widely exist among ascomycete fungi. Here we provide experimental evidence for shared function of a *col-26* ortholog in *Trichoderma reesei*. Our finding provides novel insight into how the regulation of carbon and nitrogen metabolism can be integrated in filamentous fungi by the function of COL-26 and which may aid in the rational design of fungal strains for industrial purposes.

## Introduction

Filamentous fungi are one of the primary degraders of plant biomass because of their ability to produce enzymes that break down complex polysaccharides, including cellulose, hemicellulose, and pectin in the plant cell wall and starch, which is the major storage component in plants. Starch consists of two types of polysaccharides, amylose and amylopectin. Amylose is composed of linear chains of α-1,4-linked glucose units, while amylopectin is composed of α-1,4-linked glucose polymers, with branched α-1,4-glucan connected through α-1,6 glucosidic bonds at branch points. Our understanding of starch degradation by filamentous fungi mainly comes from work in *Aspergillus spp*. (reviewed in [1]), which are industrially important producers of starch-degrading enzymes. Three types of enzymes, α-amylases, glucoamylases, and α-glucosidases, hydrolyze starch synergistically to produce glucose. α-Amylases hydrolyze α-1,4-glucan chains endolytically to produce maltose, while α -glucosidases and glucoamylases hydrolyze maltose and α -1,4-linkage exolytically from non-reducing ends to form glucose. Glucoamylases also hydrolyze α -1,6 linkages at branch connections. Recently, a new family of lytic polysaccharide monooxygenases (LPMO) active on starch was identified in *Neurospora crassa* [2]. The starch-active LPMOs together with a biological redox partner oxidatively cleave amylose, amylopectin, and starch. The expression of genes encoding amylolytic enzymes can be induced by starch and its degradation products, maltose and glucose [3–5].

In *Aspergillus spp*., expression of genes encoding amylolytic enzymes requires the transcriptional activator AmyR, a zinc binuclear cluster (Zn(II)2Cys6) DNA-binding protein belonging to the Gal4p family of transcription factors [6]. Disruption of *amyR* in *A*. *oryzae* and *A*. *nidulans* leads to significantly decreased amylolytic enzyme activities and restricted growth on starch medium [7, 8]. A similar role in starch hydrolysis was demonstrated for *amyR* homologs in *Penicillium decumbens* [9], *Fusarium verticillioides* and *F*. *graminearum* [10]. Genome sequencing of two *Trichoderma reesei* mutant strains, RUT C30 and PC-3-7, with enhanced cellulase production and resistance to carbon catabolite repression (CCR) identified SNPs in the *bglR* gene, a homolog of *amyR* [11, 12]. Although a *T*. *reesei* strain bearing a deletion of *bglR* was reported having reduced growth on maltose and glucose, further investigation on the phenotype of the Δ*bglR* mutant was not reported. Instead, Nitta *et al*. (2012) suggested that BglR regulates genes encoding β-glucosidases and belongs to a new functional transcription factor group distinguishable from AmyR based on two observations [11]. First, when induced by cellobiose, expression of some β-glucosidase genes was lower in the Δ*bglR* mutant as compared to the parental PC-3-7 strain. Second, AmyR and BglR form two separate clusters in phylogenetic analyses. However, the AmyR homologs in *F*. *graminearum* and *F*. *verticillioides (*FgART and FvART, respectively) are in the same cluster as BglR and are essential for starch utilization [10].

COL-26 is the *N*. *crassa* ortholog of BglR and was named *colonial-26* (*col-26*) for its colonial phenotype on medium containing sucrose, glucose or fructose as a sole carbon source [13, 14], suggesting COL-26 plays a role in regulating glucose metabolism. In *N*. *crassa*, COL-26 was shown to function synergistically with CRE-1, a transcription factor important for CCR and in regulating cellulase gene expression and enzyme production [14]. The Δ*col-26* mutant is also resistant to 2-deoxyglucose, suggesting it has impaired CCR.

In this study, we tested growth phenotypes of the Δ*col-26* mutant on a variety of carbon sources and determined that COL-26 is essential for maltose and starch utilization. We determined that the absence of *col-26* led to a decrease in expression of amylolytic genes. Metabolic analyses of the Δ*col-26* mutant in comparison to WT cells indicated that mis-regulation of the TCA cycle, GABA shunt, and amino acid biosynthesis occurs in the Δ*col-26* mutant. Replacing ammonium as a nitrogen source on preferred carbon sources with glutamine alleviated the growth defects of Δ*col-26* on glucose, but not on maltose medium. Our study indicates that COL-26 has an important and conserved role in the regulation of starch degradation as coordinating primary carbon and nitrogen metabolism in filamentous fungi, and provides insight for the rational design of strains for the food and biofuel industries.

## Results

### Growth phenotypes of the Δ*col-26* mutant on different carbon sources

**Fig 1:**
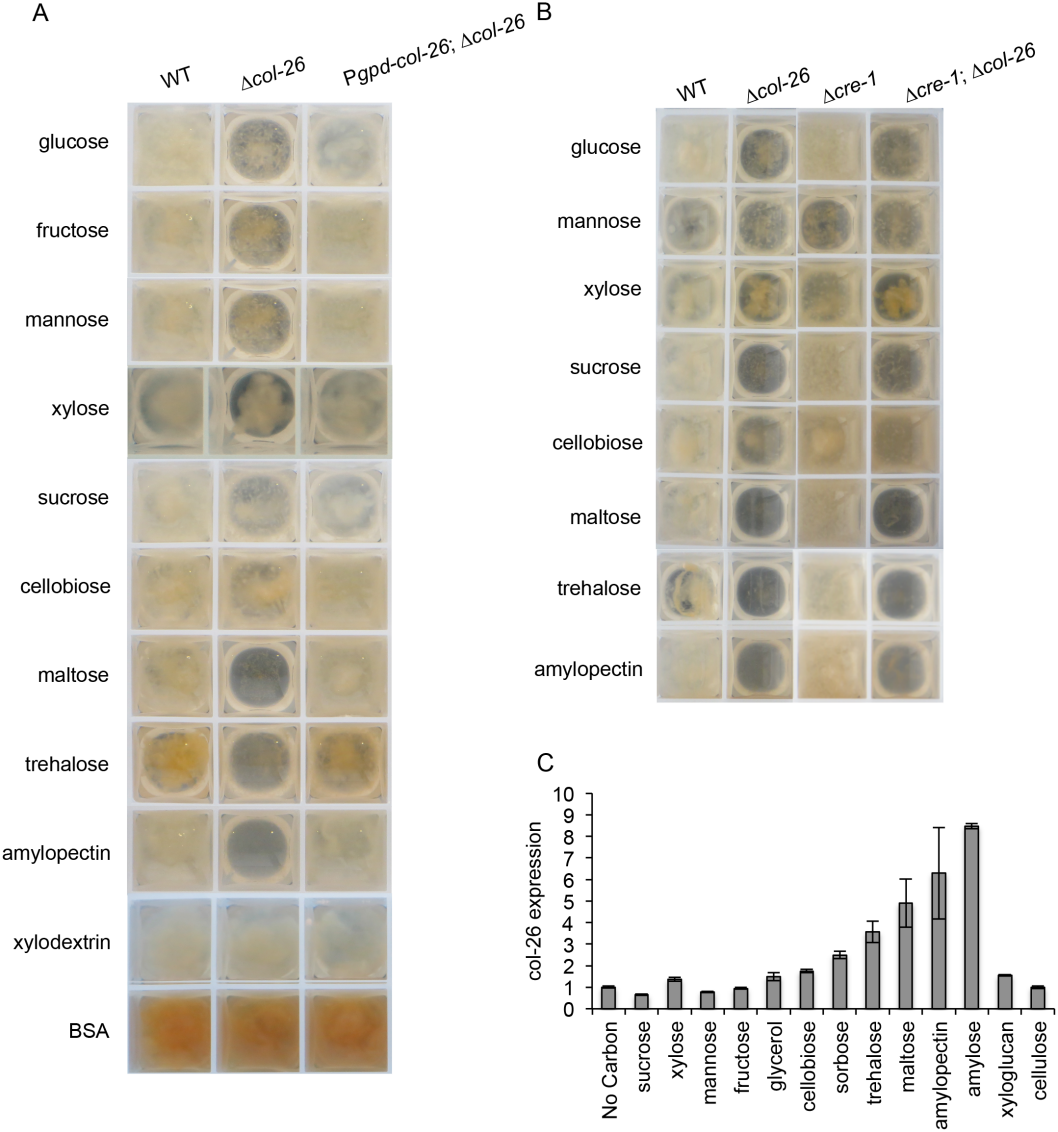
Growth of WT and *col-26* mutants in VMM with different carbon sources. (A) Growth of the wild type parental strain (FGSC 2489), the Δ*col-26* mutant and the *col-26* complemented strain (P*gpd-col26;Δcol-26*) in liquid VMM with indicated carbon source after 24 hrs of growth, except for the xylodextrin cultures, which were grown for 39 hrs. (B) Growth of the wild type parental strain (FGSC 2489), Δ*col-26*, Δ*cre-1*, and Δ*cre-1;*Δ*col-26* strain in liquid VMM with indicated carbon source after 24 hrs. (C) Fold change in expression levels of *col-26* in the WT strain when exposed to different carbon sources in switch experiments (see Materials and methods). Fold change was determined by comparing the *col-26* transcript abundance in samples grown in VMM with the indicated carbon source to the mean expression level of *col-26* in samples switched to VMM with no carbon. Transcript abundance was measured by RNA-seq experiments Error bar: standard deviation from three biological replicates.

The Δ*col-26* mutant poorly utilizes simple sugars, including glucose, fructose, and sucrose, but grows well on complex polysaccharides such as cellulose [14]. To test whether COL-26 is important for the utilization of other carbon sources, we tested the growth of the Δ*col-26* mutant on different mono-, di- or polysaccharides as a sole carbon source. As observed previously, the Δ*col-26* mutant showed reduced growth in glucose, fructose and sucrose [14], but also showed reduced growth on xylose and cellobiose and essentially no growth on maltose or trehalose (Fig 1A). On complex polysaccharides, such as xylodextrins and albumin, the Δ*col-26* mutant grew similarly to the WT parental strain. However, the Δ*col-26* mutant showed a severe growth defect on amylopectin (Fig 1A). To verify that *col-26* is causative for these growth phenotypes, we introduced a copy of the *col-26* gene under regulation of the *A*. *nidulans gpd* promoter at the *csr-1* locus in the Δ*col-26* mutant (see Materials and methods). This P*gpd-col-26;* Δ*col-26* strain showed a similar growth phenotype as the WT strain on these different carbon sources (Fig 1A). Consistent with the hypothesis that COL-26 plays a role in regulating genes encoding enzymes required for utilization of starch, trehalose and maltose, the expression level of *col-26* was induced 4 to 8 -fold by a 4-hr exposure to trehalose, maltose, amylopectin and amylose (Fig 1C).

A genetic interaction between *cre-1* and *col-26* was revealed in the regulation of cellulase production; increased expression levels of *cre-1* was observed in the Δ*col-26* mutant [14]. This observation suggested that mis-regulation of *cre-1* (and thus inappropriate triggering of CCR) may play a role in the growth phenotype of the Δ*col-26* mutant. To test this hypothesis, we examined the growth phenotype of the Δ*cre-1;* Δ*col-26* double mutant as compared to the WT strain and the Δ*col-26* and Δ*cre-1* single mutants on a variety of carbon sources. The Δ*cre-1;* Δ*col-26* mutant grew similarly to the Δ*col-26* mutant when glucose, xylose, sucrose, cellobiose, maltose, trehalose, or amylopectin was used as the sole carbon source (Fig 1B), indicating that the mis-regulation of *cre-1* expression was not causative for the poor growth phenotype observed in the Δ*col-26* mutant.

### Transcriptional profiling of the wild type strain on starch components

Neurospora has long been known to be a starch utilizer ever since its discovery over 170 years ago on contaminated bread in a French Bakery [15]. Although mutants deficient for the utilization of starch (*sor-4*, *gla-1* and *gla-2*) have been identified [16], how *N*. *crassa* transcriptionally responds to starch in its environment has not been previously investigated. To provide systematic data on expression changes in response to defined polysaccharide constituents of starch, we performed transcriptional profiling of WT cells exposed to Vogel’s minimal medium (VMM) [17] containing amylose or amylopectin as the sole carbon source (1% w/v) and WT cells exposed to VMM containing maltose as the sole carbon source (2% w/v). RNA-seq data from *N*. *crassa* cultures exposed to VMM with no carbon (NC) or VMM with 2% (w/v) sucrose were included as controls.

**Fig 2:**
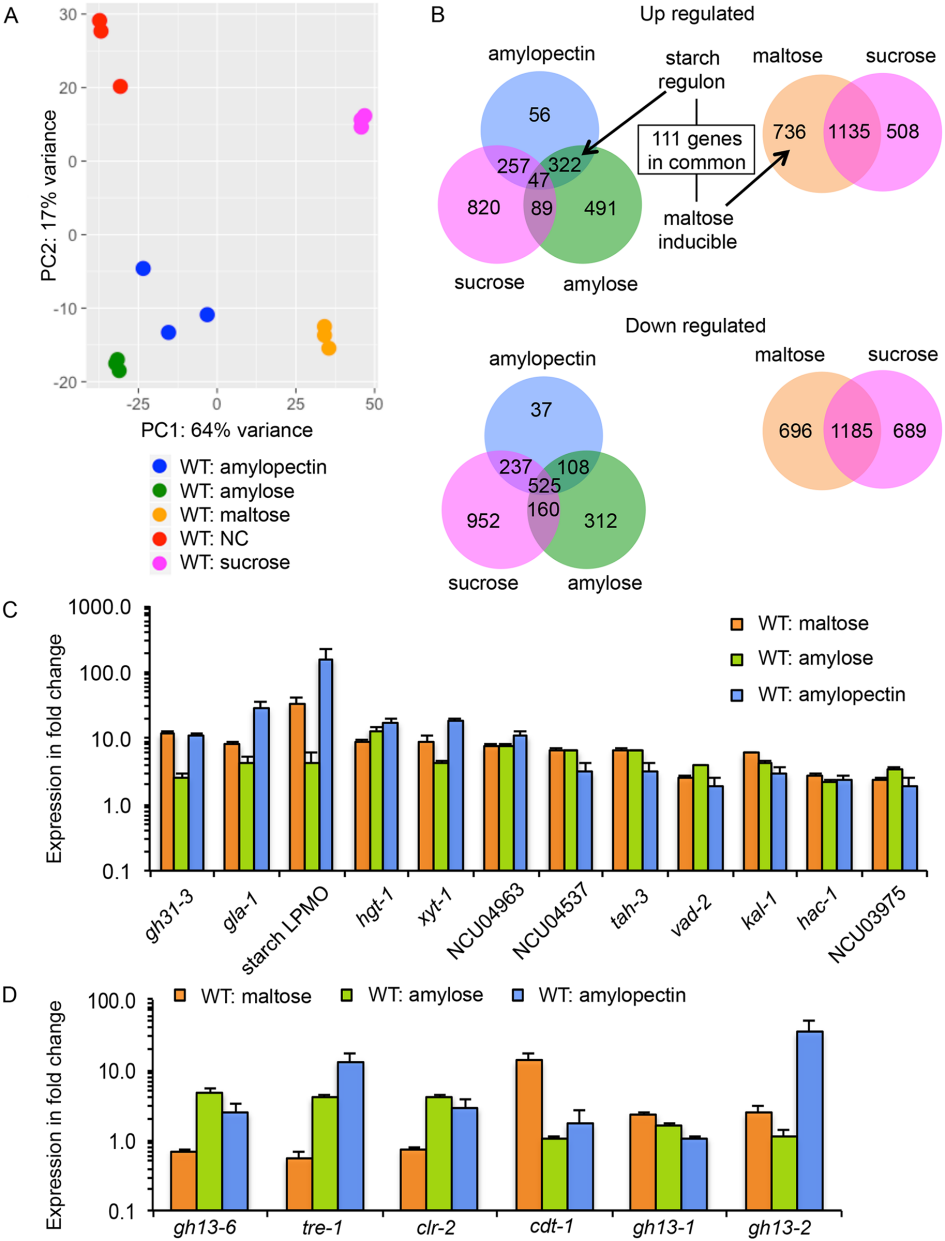
Transcriptome of WT strain grown in starch components. (A) Principal component analysis (PCA) of RNA-seq data from the WT strain (FGSC 2489) exposed to different carbon sources. NC: VMM with no carbon. (B) Venn diagram of differentially expressed genes in WT cells exposed to sucrose, maltose, amylose and amylopectin. (C) Fold change in expression levels in WT cells of genes induced by all three starch components, maltose, amylose and amylopectin. (D) Fold change in expression levels of genes significantly induced by starch polysaccharides versus by maltose. Fold change is the relative transcript abundance compared to WT grown in VMM-NC. Error bar: standard deviation from three biological replicates.

The fifteen sets of RNA-seq data were first evaluated using principle component analysis (PCA). Biological replicate samples from the same carbon condition clustered tightly (Fig 2A). Expression patterns from cultures exposed to amylose and amylopectin also clustered closer to each other than to the NC, maltose and sucrose samples, suggesting a common transcriptional response in *N*. *crassa* upon exposure to polysaccharides of starch. Additionally, expression patterns from cultures exposed to maltose were distant from those exposed to sucrose in the PCA plot, suggesting substantial transcriptional changes specifically induced by maltose.

Pairwise comparison between the transcriptome of WT cells exposed to amylose or to amylopectin compared to that of VMM-NC revealed genes with differential expression levels (fold change greater than 2, and false discovery rate (FDR)-corrected p value below 0.01). After subtracting genes that were also differentially induced or repressed in sucrose as compared to VMM-NC, we identified 322 genes that increased in expression level in WT cells upon exposure to amylose/amylopectin and 108 genes that showed reduced expression levels in amylose/amylopectin (Fig 2B; S1 Table, Sheet 1). We name this 322-gene set the “starch regulon”. Indeed, the only overrepresented KEGG pathway in this set of 322 genes was starch and sucrose metabolism (adjusted p-value: 4.8e-3). No KEGG pathway was overrepresented in the 108 reduced expression gene set. Analyses of RNA-seq data from WT on maltose revealed a set of 1871 genes that increased in expression level and 1881 genes that decreased in expression level compared to data from WT on NC. After subtracting genes that were similarly regulated by sucrose, we identified 736 genes with increased expression level and 696 genes with decreased expression level in WT cells on maltose medium (Fig 2B; S1 Table, Sheet 2). The maltose-inducible gene set was enriched in genes from functional categories of biogenesis of cell wall, perception of nutrient and nutritional adaptation, and electron transport and membrane-associated energy conservation. Additionally, the maltose-inducible gene set overlapped the starch regulon by 111 genes (S1 Table, Sheet 3). A search in the Carbohydrate Active Enzymes (CAZyme) database (http://www.cazy.org/) [18] revealed that 7 of the 111 genes were predicted to act on carbohydrates. Three of them, NCU04674 (*gh31-3*), NCU01517 (*gla-1*), and NCU08746 have annotated functions in degrading starch. *gh31-3* encodes a α-glucosidase, *gla-1* encodes a glucoamylase and NCU08746 encodes a lytic polysaccharide monoxygenase that acts on starch [2] (Fig 2C). A BLASTP search of the transporter classification databases (TCDB) (http://www.tcdb.org/) with cut-off value less than 1e-20 identified 14 genes likely encoding transporters. Four of them, *hgt-1* (NCU10021), NCU05627, NCU04963, and NCU04537 are annotated as sugar transporter genes (Fig 2C). *hgt-1* shows high affinity glucose transport activity [19], while NCU05627 (*xyt-1*) has xylose transporting activity [20]. The transport substrates of NCU04963 and NCU04537 remain to be determined. There are also 5 TF genes induced by all three starch components, *tah-3*, *vad-2*, *kal-1*, *hac-1*, and NCU03975 (Fig 2C). *tah-3* was found to be required for tolerance to a harsh plasma environment [21]. For VAD-2 and KAL-1, a role in nutrient metabolism or sensing has been proposed [13]. HAC-1 is involved in the unfolded protein response and is necessary for growth on cellulose, but not hemicellulose in *N*. *crassa* [22].

Genes in the starch-regulon, but that were not in the maltose-inducible gene set, included *gh13-6*, *tre-1*, and *clr-2* (Fig 2D). *gh13-6* encodes an α-amylase, *tre-1* encodes a trehalase, and CLR-2 is the major transcriptional regulator of cellulase genes in *N*. *crassa* and is essential for the utilization of cellulose [23–25]. Among genes significantly induced by maltose, but not by starch polysaccharides were NCU00801 (*cdt-1*) and NCU12154 (Fig 2D). CDT-1 is a cellodextrin transporter and NCU12154 was annotated as maltose permease. The latter shows low homology to the yeast maltose permease (P53048; TCDB database). Interestingly, the α-amylase gene (*gh13-6*) in the starch regulon was not induced by maltose (Fig 2D). Instead, two other α-amylase genes (NCU09805 *gh13-1* and NCU08131 *gh13-2*) were significantly induced (Fig 2D).

### Comparative transcriptional analysis of WT and the *col-26* mutant

**Fig 3:**
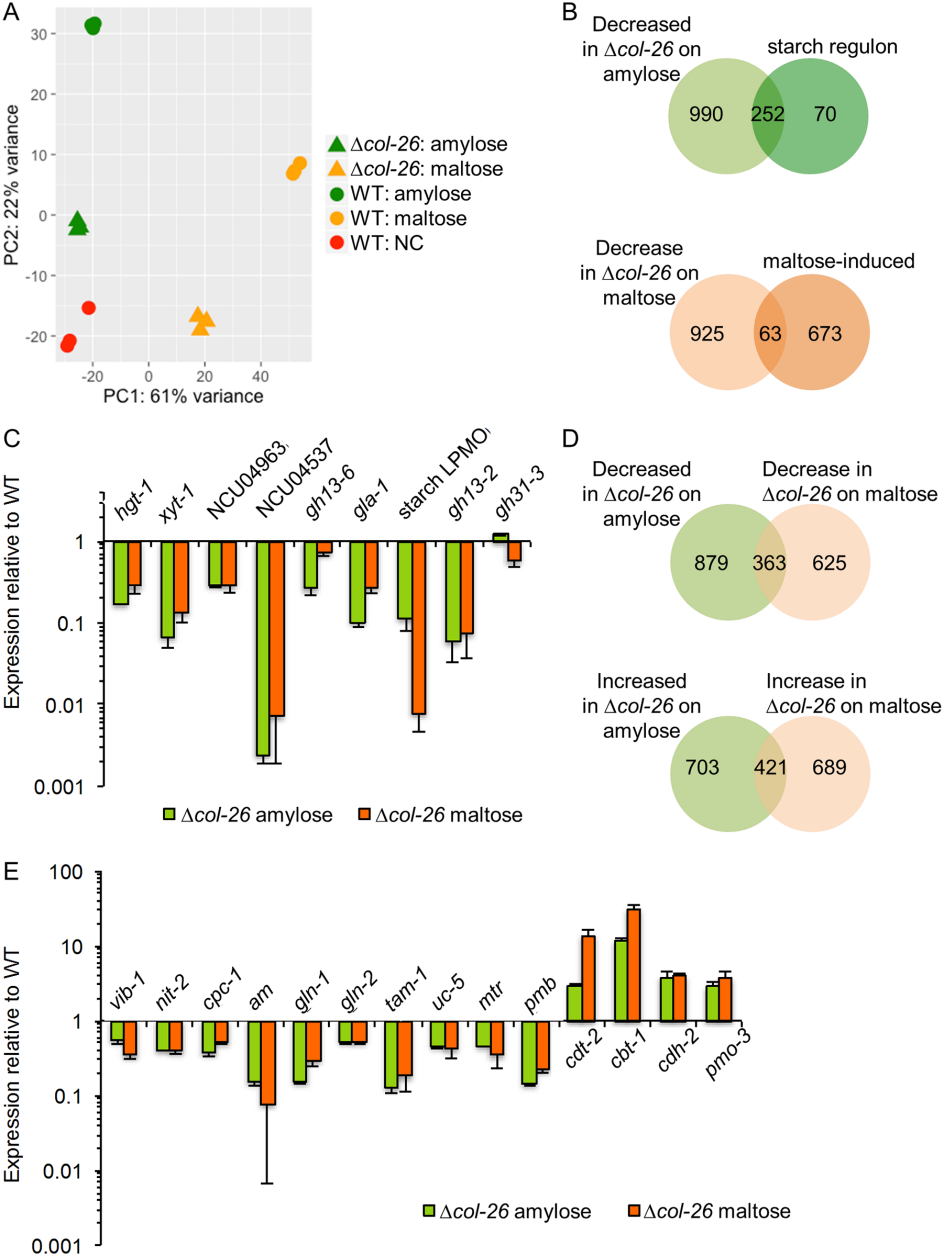
Transcriptome of WT and Δ*col-26* on maltose and amylose and genes impacted by the *col-26* deletion. (A) Principal component analysis (PCA) of RNA-seq data from the WT and Δ*col-26* strains grown on either amylose or maltose. (B) Venn diagram of genes down-regulated in the Δ*col-26* mutant on amylose and maltose compared to the starch regulon and the maltose-inducible gene set identified in WT cells. C) Fold change in expression levels of genes induced by all three starch components (amylose, amylopectin and maltose), but down regulated in the Δ*col-26* mutant. (D) Venn diagram of differentially expressed genes in the Δ*col-26* mutant on amylose as compared to maltose. (E) Fold change in expression of selected *col-26-*dependent genes. Error bar: standard deviation from three biological replicates.

The Δ*col-26* mutant failed to grow on maltose and amylopectin (Fig 1A). To investigate the functions of COL-26 required for utilization of these substrates, we evaluated transcriptional changes in the Δ*col-26* mutant when switched to medium containing amylose or maltose under identical conditions as with the WT parental strain (see above). RNA-seq data from the WT and Δ*col-26* biological replicates were subjected to PCA analysis and data from the same strain grown under the same growth conditions clustered together (Fig 3A). On the PCA plot, data from the Δ*col-26* mutant exposed to amylose and data from the Δ*col-26* mutant exposed to maltose did not cluster.

Under amylose conditions, the expression level of 1242 genes was significantly lower in the Δ*col-26* mutant, while the expression level of 1124 genes increased (Fig 3B, S2 Table). Strikingly, 252 genes out of the 322-gene starch regulon gene set (78%) were down regulated in the Δ*col-26* mutant (Fig 3B), including three of the four amylolytic genes, *gla-1*, the starch-active LPMO (NCU08746), *gh13-6* and 19 transporter genes including *hgt-1*, *xyt-1*, NCU04963, and NCU04537 (Fig 3C). Seventeen TF genes in the starch regulon were also down regulated in the Δ*col-26* mutant, including *tah-1*, *tah-3*, *vad-2*, *ada-5*, and *kal-1* (S2 Table, sheet 1). The majority of the remaining 70 starch-regulon genes (41 genes) whose expression levels were not affected by the *col-26* deletion were annotated as hypothetical. These data indicate that COL-26 is a major regulator of the starch regulon of *N*. *crassa*. The down regulation of expression of the starch regulon genes by deletion of *col-26* is consistent with the growth defect observed in the Δ*col-26* mutant on starch polysaccharides (Fig 1A).

Under maltose conditions, the expression levels of 1110 genes were significantly increased in Δ*col-26* mutant, while the expression levels of 988 genes decreased (Fig 3B and S2 Table, sheet 2). The three amylolytic genes, i.e., *gla-1*, *gh13-2*, and the starch LPMO (NCU08746) and the four sugar transporter genes, *hgt-1*, *xyt-1*, NCU04963, and NCU04537 were members of the down-regulated gene set (Fig 3C). This down-regulated gene set also included additional 100 transporter genes, many from the Mitochondrial Protein Translocase (MPT) family, the Nuclear mRNA Exporter (mRNA-E) family, the Mitochondrial Carrier (MC) family, and the Major Facilitator Superfamily (MFS) (S2 Table). The down-regulated 988-gene set in the Δ*col-26* mutant only overlapped the 736 maltose-inducible set in WT cells by 63 genes (Fig 3B). Directly comparing the amylose and maltose RNA-seq data between the WT and the Δ*col-26* mutant showed that 363 genes were down regulated and 421 genes were up regulated in absence of *col-26*. We named the 363 genes as the “COL-26-dependent gene set” and the 421 genes as the “COL-26-reduced expression gene set” (S3 Table, Fig 3D). Only 33 genes in the COL-26-dependent gene set (less than 10%) were induced in WT cells by exposure to maltose, amylose, or amylopectin.

### Analyses of the COL-26-dependent and reduced expression gene sets

For the COL-26-dependent gene set, a functional enrichment analysis using FunCat [26] showed that transcription and protein synthesis were overrepresented, including rRNA processing, where 79 of 198 genes in this category were identified as being COL-26 dependent (p = 9e-52). Genes from categories such as RNA binding functions, ribosome biogenesis, rRNA modification, mRNA synthesis and mitochondrial transport were also enriched (p = 3e-17, 2e-13, 3e-9, 2e-7, and 1e-2 respectively). The COL-26-dependent gene set contained 6 TF genes besides *col-26*. Three were annotated to be hypothetical, and the other three were *vib-1* (NCU03725), *nit-2* (NCU09068), and *cpc-1* (NCU04050) (Fig 3E). VIB-1 (vegetative incompatibility block-1) is required for extracellular protease secretion in response to both carbon and nitrogen starvation [27] and for the utilization of cellulose [14]. The *cpc-1* gene (*cross-pathway control*-1) is the ortholog of *S*. *cerevisiae GCN4*, and is required in *N*. *crassa* for the expression of many amino acid biosynthetic genes in response to amino acid starvation [28–30]. Ten genes in CPC-1 regulon [30] were also found in the COL-26-dependent gene set (S3 Table). The *nit-2* gene (nitrate nonutilizer-2) is the major regulatory transcription factor in *N*. *crassa* regulating nitrogen catabolism and is critical for utilization of nitrate, nitrite, purines, and most amino acids as a nitrogen source (reviewed in [31]). Also in this set were genes encoding catabolic enzymes in nitrogen metabolism and amino acid synthesis such as *am*, which encodes the NADP-glutamate dehydrogenase (NADP-GDH), *gln-1* (NCU06724) and *gln-2* (NCU04856), both of which encode glutamine synthases. Several transporters in this set are also predicted to be involved in nitrogen and amino acid assimilation, including *uc-5* (NCU07334), *mtr* (NCU06619), *pmb* (NCU05168) and *tam-1* (NCU03257). *uc-5* encodes a uracil permease [32]. The *mtr* mutant is defective in transport of neutral aliphatic and aromatic amino acids. The *pmb* mutant is defective in basic L-amino acid transport and has reduced uptake of L-arginine, L-lysine, and L-histidine [16] and *tam-1* encodes a predicted ammonium transporter.

We also compared the COL-26-dependent gene set to the set of genes that showed reduced expression levels in WT cells in carbon-free medium as compared to WT on maltose or on amylose to reflect the effects of carbon starvation under these two conditions. This comparison revealed that 291 of the 363 COL-26-dependent genes also showed reduced expression in WT cells when no carbon source was available (S3 Table), including *vib-1*, *nit-2*, *uc-5*, *mtr*, *pmb*, *am*, and 5 of the 10 CPC-1 regulon genes. However, *cpc-1*, *gln-1* and *gln-2* were not among these 291 genes.

The COL-26-reduced expression gene set was enriched with genes in the functional categories of non-vesicular cellular import (p = 7e-8), secondary metabolism (p = 4e-6), degradation or biosynthesis of phenylalanine (p = 2e-5), allantoin and allantoate transport (p = 2e-7), polysaccharide metabolism (p = 2e-6), and C-compound and carbohydrate transport (p = 9e-6). This gene set also included 39 transporter genes, 7 TF genes, and 26 CAZyme genes (S3 Table). All predicted TF genes in this set have no assigned function. The majority of the transporter genes (25 of 39) belong to the MFS family, but only two, *cdt-2* and *cbt-1* (NCU08114 and NCU05853) have been characterized (Fig 3E). CDT-2 transports cellodextrins and xylodextrins [33–35], while CBT-1 has transporting activity for cellobionic acid [36, 37]. The 26 CAZymes are from 21 CAZyme families, and two of them, a cellulose LPMO gene *pmo-3* (NCU07898) and a cellobiose dehydrogenase gene *cdh-2* (NCU05923) have been characterized in *N*. *crassa* [38, 39]. Of these 421 genes, 195 were also de-repressed in WT cells under carbon-free conditions relative to WT on maltose or amylose.

### Growth defects of the Δ*col-26* mutant are restored by the substitution of nitrate/ammonium with glutamine as the sole nitrogen source

COL-26 was essential for the utilization of starch components and was essential for expression of a large fraction of genes associated with utilization of starch in WT cells (Figs 1 and 3B). However, its growth defect on preferred carbon sources was unique, as other mutants, such as Δ*clr-1* and Δ*clr-2* are unable to grow on cellulose, but have WT growth rates on preferred carbon sources [23]. Our observation of the down regulation of *tam-1*, *am*, *gln-1* and *gln-2* genes (Fig 3E) in the Δ*col-26* mutant and the fact that loss-of-function of glutamine synthase renders *N*. *crassa* dependent upon glutamine for normal growth [40] prompted us to test whether the reduced growth of the Δ*col-26* mutant in VMM-glucose was due to impaired nitrogen metabolism.

**Fig 4:**
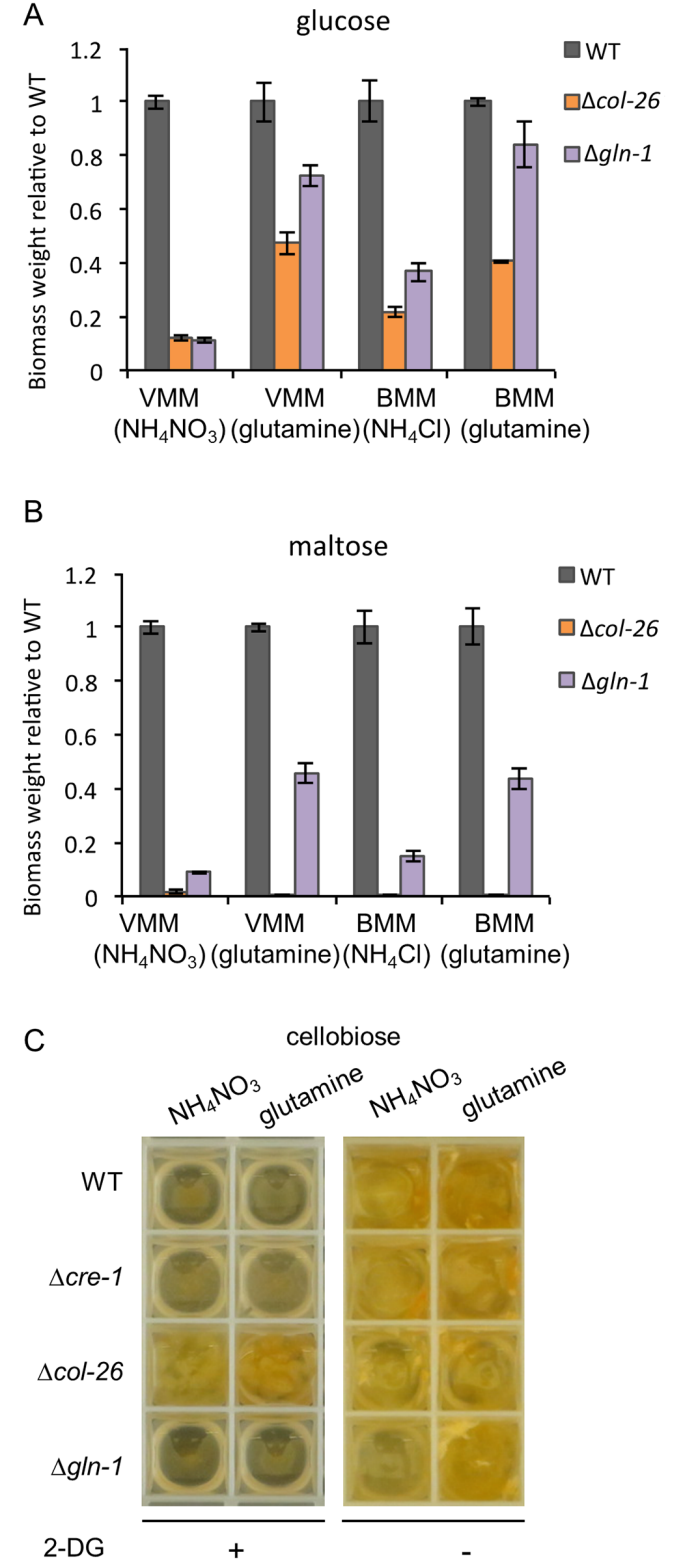
Effect of glutamine replacement as a nitrogen source on growth of WT, Δ*col-26* and Δ*gln-1* strains on glucose or maltose as a sole carbon source. (A) Fungal biomass accumulation in WT and mutant strains was measured after 24 hrs of growth on indicated media with glucose as the sole carbon source. (B) Fungal biomass accumulation in WT and mutant strains was measured after 24 hrs of growth on indicated media with maltose as the sole carbon source. VMM(NH_4_NO_3_): Vogel’s minimal medium [17] with ammonium nitrate as the nitrogen source. VMM(glutamine): Vogel’s minimal medium with glutamine (25 mM) as the nitrogen source. BMM(NH_4_Cl): Bird’s minimal medium [41] with ammonium as the sole nitrogen source. BMM(glutamine): Bird’s minimal medium with glutamine (25 mM) as the nitrogen source. (C) Growth of WT, Δ*cre-1*, Δ*col-26* and Δ*gln-1* strains in VMM with either NH_4_NO_3_ or glutamine as the sole nitrogen source with or without 2-DG (0.2%) supplementation (+ and -, respectively) after 48 hrs. Cellobiose (2% w/v) was used as the carbon source.

To test this hypothesis, we examined growth of the Δ*col-26* mutant in VMM where ammonium nitrate was replaced by glutamine, VMM(Gln). Bird’s minimal medium (BMM) [41] was also used for assessing the effect of glutamine substitution, BMM(Gln); BMM(NH_4_Cl) has ammonium chloride as the sole nitrogen source. The carbon source for both VMM(NH_4_NO_3_) and BMM(NH_4_Cl) was glucose; the Δ*col-26* mutant showed decreased growth on VMM(NH_4_NO_3_)-glucose (Fig 1A). Mycelial biomass from 24-hr cultures of the WT and Δ*col-26* strains, as well as a glutamine synthetase mutant (Δ*gln-1*) [16] were compared (Fig 4A). In VMM(NH_4_NO_3_) with 2% (w/v) glucose as the carbon source, both Δ*col-26* and Δ*gln-1* grew poorly, reaching only 11~ 12% of WT biomass. Both mutants grew much better in VMM(Gln), with Δ*col-26* and Δ*gln-1* reaching 47% and 72% of WT biomass, respectively (Fig 4A). Similar rescue effects of glutamine were observed in the Δ*col-26* and Δ*gln-1* mutants in BMM(Gln) as compared to that in BMM(NH_4_Cl) (Fig 4A). Substitution of glutamine for ammonia also partially rescued the growth of the Δ*gln-1* mutant on maltose or amylopectin, but failed to rescue the growth of the Δ*col-26* mutant under the same conditions (Fig 4B). The distinctive rescue effect of glutamine in the Δ*col-26* mutant on medium with glucose versus medium with maltose argues for glutamine being used as a nitrogen source rather than a carbon source. To test this hypothesis, WT, and the Δ*col-26*, Pgpd-*col-26* and Δ*gln-1* strains were grown on VMM with either glutamine, glutamate, arginine or proline as both the carbon and nitrogen source or as the carbon source with ammonium nitrate as the nitrogen source (S1 Fig); none of the strains efficiently utilized these amino acids as a carbon source, with only minimal mycelial biomass observed after 9 days of growth (S1 Fig).

The Δ*col-26* and *Δcre-1* mutants are resistant to 2-deoxyglucose (2-DG), a glucose analog that cannot be metabolized but is able to trigger CCR [14]. It is often used to select for, or evaluate, impairment of CCR or glucose repression in filamentous fungi [42, 43]. As Δ*col-26* grows extremely slowly in VMM(NH_4_NO_3_) with glucose, we hypothesized that its insensitivity to 2-DG may be a result of its mis-regulation of carbon/nitrogen metabolism. Since the growth of the Δ*col-26* mutant in VMM(Gln) was enhanced, we evaluated whether this restoration in growth rescued the sensitivity to 2-DG in the Δ*col-26* mutant. We used cellobiose as the sole carbon source, as the Δ*col-26* mutant grows better under these conditions (Fig 1A). The WT, Δ*cre-1*, Δ*col-26*, and Δ*gln-1* strains were grown in VMM-2% (w/v) cellobiose with or without 0.2%(w/v) 2-DG, with either NH_4_NO_3_ or glutamine as the nitrogen source. The Δ*col-26* mutant showed a clear resistance to 2-DG, independently of whether NH_4_NO_3_ or glutamine was used as the nitrogen source (Fig 4C). These data indicate that Δ*col-26* resistance to 2-DG inhibition (and thus impaired COL-26-mediated CCR) remains independent of the nitrogen source.

### Comparative metabolite analysis of WT and the Δ*col-26* mutant

**Fig 5:**
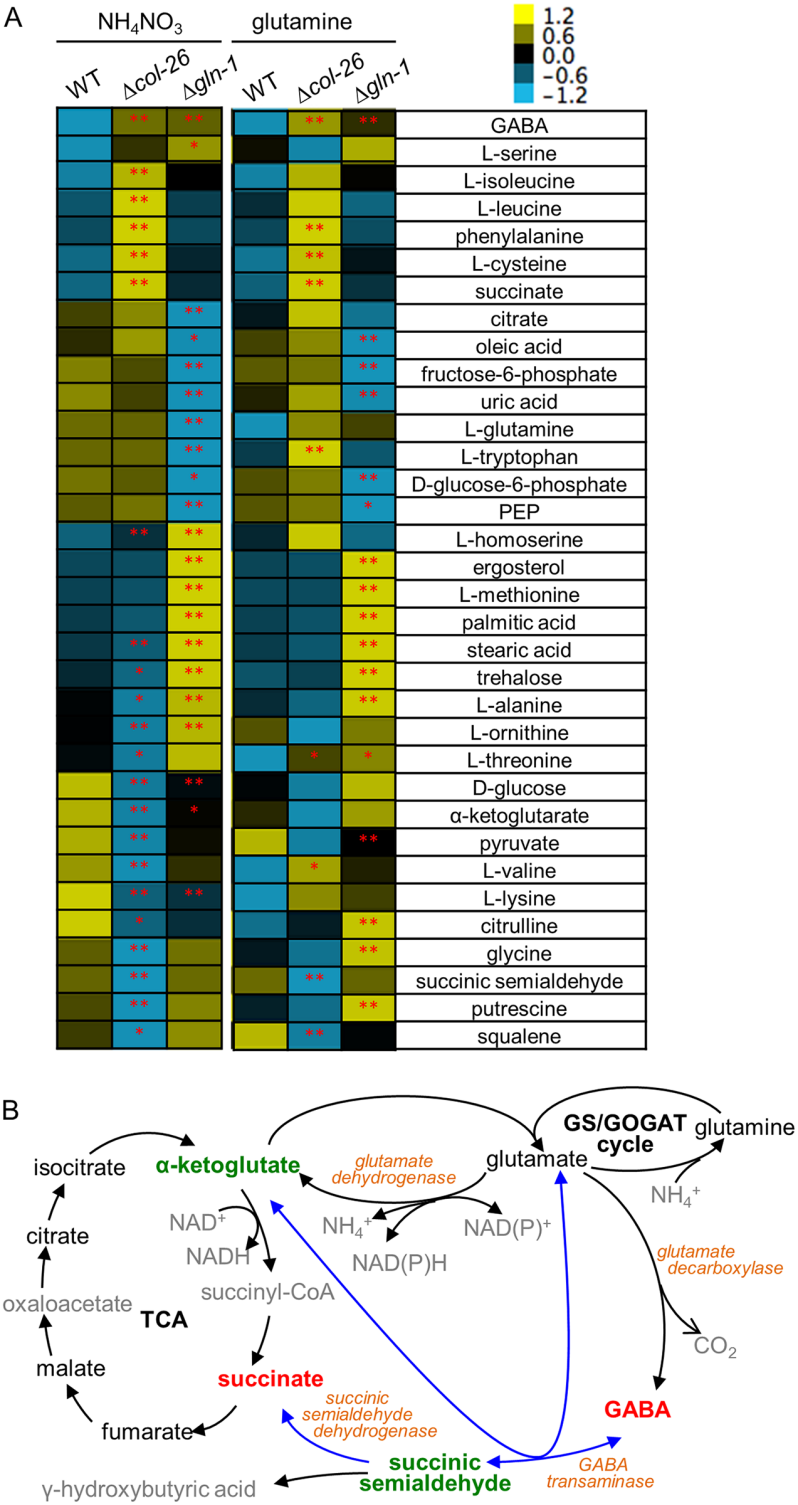
Metabolite profiling of WT and Δ*col-26* strains. (A) Heat map of metabolites whose levels were significantly different between the WT parental strain and the Δ*col-26* or Δ*gln-1* mutant were grouped by hierarchical clustering based on mean level of biological replicates from VMM with glucose and either ammonium nitrate or glutamine as the sole nitrogen source. Single asterisk: (0.05 < p < 0.1). Double asterisk: (P <0.05). (B) TCA and the GABA shunt pathways. Black: metabolites detected and measured in our experiments. Grey: metabolites not detected or measured. Red: metabolites showing higher accumulation in the Δ*col-26* mutant as compared to the WT parental strain. Green: metabolites showing significantly lower accumulation in the Δ*col-26* mutant relative to the WT strain. Blue arrows: the GABA shunt in the mitochondria.

To further understand the changes in primary carbon, nitrogen, and amino acid metabolism in the Δ*col-26* mutant relative to the WT strain, we profiled 45 intracellular metabolites from WT, Δ*col-26* and Δ*gln-1* strains grown on VMM (NH_4_NO_3_) or VMM(Gln) with glucose as the carbon source (S4 Table). Strains were first grown in VMM(NH_4_NO_3_)-cellobiose (2% w/v) to accumulate fungal biomass, then switched to VMM(NH_4_NO_3_)-NC for 18 hrs and subsequently grown in VMM(NH_4_NO_3_) or VMM(Gln) with glucose (2% w/v) for an additional 5.5 hrs. Intracellular metabolites were extracted and subjected to analyses using gas chromatography coupled to mass spectrometry (GC-MS) augmented with liquid chromatography coupled to tandem mass spectrometry (LC-MS/MS); normalized abundances of metabolites were compared between WT and the mutants. Relative quantitative analysis showed that 17 metabolites were significantly different between WT and the mutants (p < 0.05) (Fig 5A). However, surprisingly little similarity in metabolite profile was observed when the Δ*gln-1* and Δ*col-26* mutants were compared.

In the Δ*gln-1* mutant, glutamine levels were significantly lower than WT when grown in VMM(NH_4_NO_3_), but the intracellular glutamine deficiency was rescued by growth on VMM(Gln) media (Fig 5A). However, although the growth defect of the Δ*col-26* mutant on glucose was partially alleviated when grown on VMM(Gln) media, the intracellular glutamine levels in the mutant were similar to that of WT grown on either VMM(NH_4_NO_3_) or VMM(Gln) (Fig 5A). Instead, the Δ*col-26* mutant accumulated high levels of four metabolites under both conditions: 4-aminobutanoic acid (GABA), phenylalanine, cysteine and succinate and was deficient in three metabolites: valine, threonine and succinic semialdehyde. Only the high level of GABA and the low level of lysine were shared phenotypes between the Δ*col-26* and the Δ*gln-1* mutant (Fig 5A). GABA and succinic semialdehyde are two intermediate metabolites in the GABA shunt, a metabolic pathway that bypasses two enzymatic steps of the TCA cycle to produce succinate from α-ketoglutarate via glutamate (Fig 5B). As the GABA shunt links primary nitrogen and carbon metabolism, the abnormal level of these intermediates suggests a mis-regulation of primary carbon and nitrogen metabolism occurs in the Δ*col-26* mutant. The levels of several amino acids showed a difference between the Δ*col-26* mutant and WT grown on VMM(NH_4_NO_3_), including homoserine, valine, lysine and threonine.

### Functional conservation of COL-26 in ascomycete species

**Fig 6:**
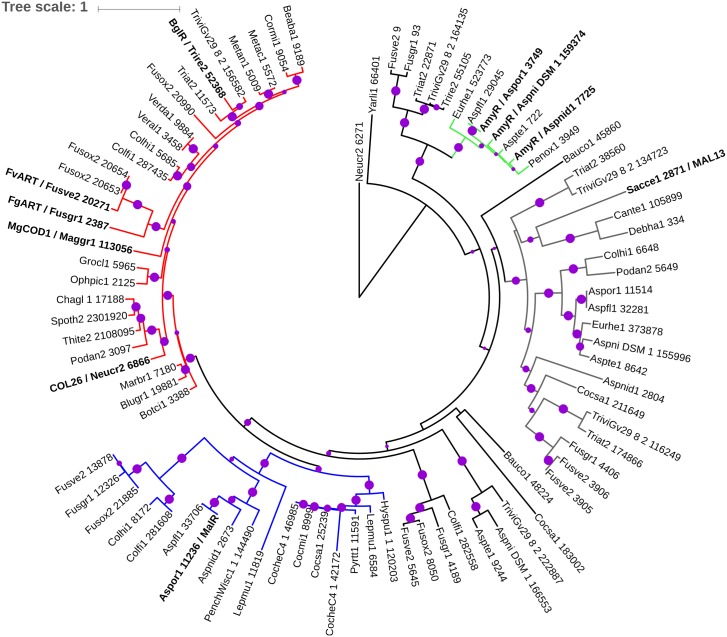
Phylogenetic analysis of COL-26. Phylogenetic tree of 86 COL-26 homologs identified in the genomes of 44 ascomycete species. The tree is rooted with Neucr2 6271/CLR2 as an outgroup. Bootstrap values from 30 to 100 are shown as purple circles with the smallest size representing 30 and the biggest size representing 100. Homologs with biological functions reported in literatures are in bold. Red: clade contains COL-26 from *N*. *crassa*, BglR from *T*. *reesei*, FgART and FvART from *F*. *graminearum* and *F*. *verticillioides*, and MgCOD1 from *M*. *grisea*. Blue: clade with MalR from *A*. *oryzae*. Green: clade with AmyR from *Aspergillus sp*. Name of homologs are abbreviated with the fungal portal name from Mycocosm (JGI) [74] followed by corresponding protein ID. Full names of the fungal species and protein ID numbers are in S5 Table.

In filamentous ascomycete fungi, COL-26, ART, and AmyR are conserved in their functions in regulating starch degradation [7, 8, 10](this study). We further demonstrated critical functions of COL-26 in integrating nitrogen and carbon metabolism, a role not previously reported for AmyR/BglR/ART orthologs in other fungi. Although phylogenetic analyses have been performed to infer functional conservation of these homologs [10, 11], either a single homolog per fungal genome was chosen or homologs from very few model organisms were included in the analyses. Our search for *col-26* homologs in 44 fungal species within the Ascomycota using BLASTP with cut-off E value of e^-20^ revealed that many fungi have more than one predicted *col-26* homolog and that the number of *col-26* homologs varies within each species (S5 Table). For example, some *Fusarium* species and *Trichoderma* species have 5 or 6 *col-26* homologs, while other species such as *Metarhizium spp*., *Verticillium spp*., *Myceliophthora thermophile*, *Thielavia terrestris*, *Chaetomium globosum*, *Cordyceps militaris*, and *Beauveria bassiana*, each have only one homolog of *col-26*. Three *Aspergillus spp*. have 3 *col-26* homologs, including *amyR*, but *amyR* from both *A*. *oryzae* and *A*. *nidulans* was not the best hit by *col-26*. In order to gain a broader view regarding functional conservation of the *col-26* homologs, we constructed a phylogenetic tree of the 86 COL-26 protein sequences using a Maximum Likelihood algorithm. CLR-2 (NCU08114; also identified as Neucr2 6271 in Mycocosm) was used as outgroup to root the tree (Fig 6). Although two COL-26 homologs exist in *T*. *reesei* (BglR/Trire2 52368 and Trire2 55109), only BglR was within the same clade as COL-26. Similarly, although *F*. *graminearum* and *F*. *verticillioides* possess 5 and 6 homologs of COL-26 respectively, only FgART and FvART were in the same clade as COL-26 and BglR. The genome of *Magnaporthe oryzae* (also called *Magnaporthe grisea)* has a single COL-26 homolog, named MoCOD1 [44]. Interestingly, the Δ*Mocod1* mutant showed significant growth reduction on glucose and maltose-containing medium but not on starch-containing medium, while the Δ*FgART1* mutant displayed a severe growth defect on glucose and starch-containing medium, but not on maltose-containing medium [10, 44]. AmyR from *A*. *oryzae*, *A*. *niger*, and *A*. *nidulans* together with two COL-26 homologs from *A*. *flavus* and *A*. *terreus*, respectively, form a clade distant from the COL-26 clade, while a MalR (AO90038000235) from *A*. *oryzae* and two homologs from *A*. *flavus* and *A*. *nidulans*, respectively, are in a clade more closely aligned to the COL-26 clade. Although AmyR is reported to be required for growth on both starch and maltose in *A*. *nidulans* [8], *A*. *oryzae* largely relies on MalR for growth on maltose [45].

**Fig 7:**
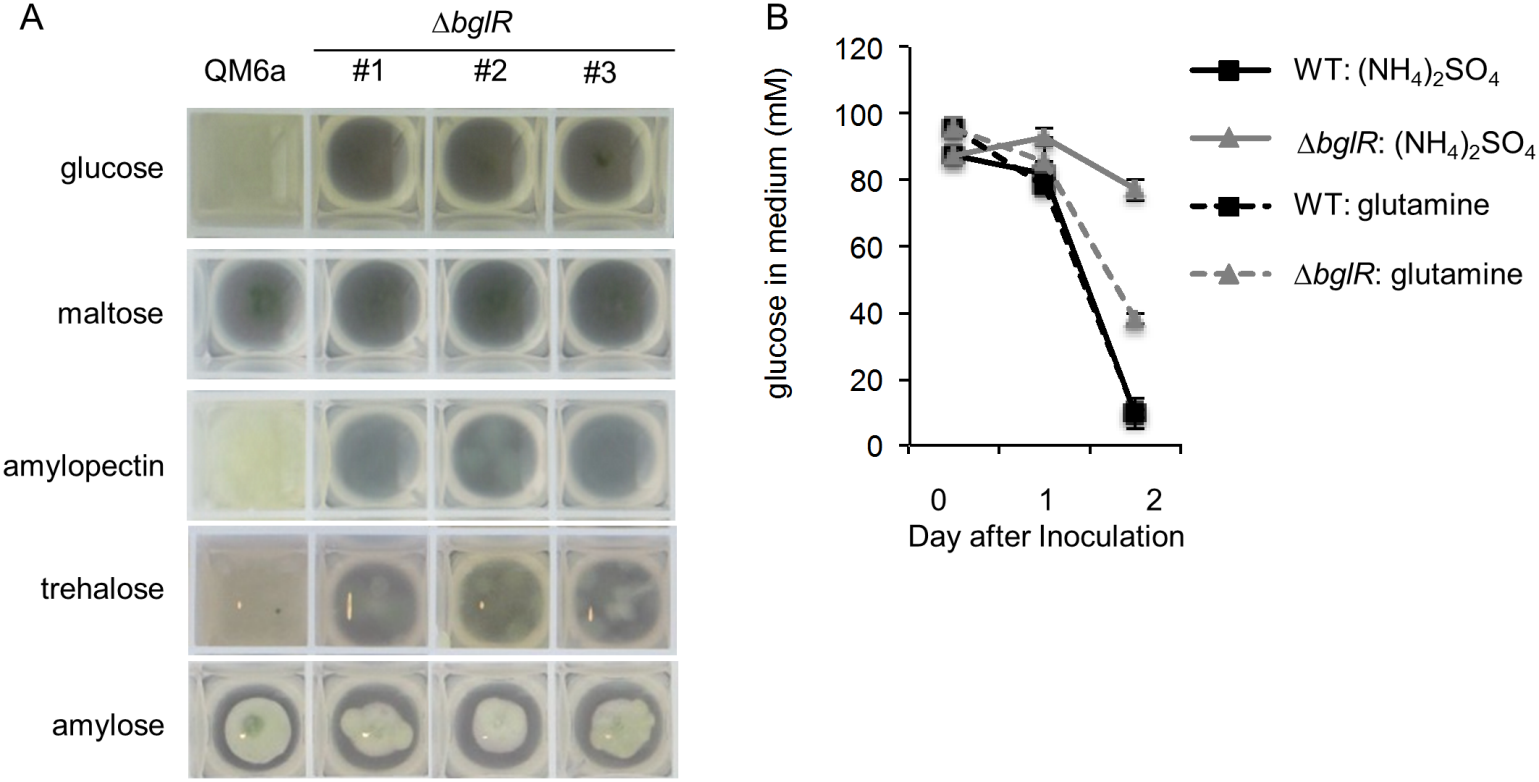
Phenotype of *T*. *reesei* Δ*bglR* mutant. (A) Growth phenotype of the *T*. *reesei* Δ*bglR* mutants grown for 44 hrs in MM-glucose, -maltose and –amylopectin and for 96 hrs in MM-trehalose and -amylose. QM6a: WT. #1, #2, #3 are three independent Δ*bglR* mutants. (B) Measurements of glucose reduction in media to infer glucose consumption rates of WT and Δ*bglR* mutant when ammonium or glutamine was used as the nitrogen source. Error bar: standard deviation from three biological replicates.

Our phylogenetic tree indicated that BglR is the closest *T*. *reesei* homolog of COL-26. Reduced growth of the Δ*bglR* mutant on maltose has been reported [11]. To test if BglR functions similarly to COL-26, we replaced the endogenous *bglR* coding sequence in *T*. *reesei* with the *pyr4* gene in a Δ*pyr4* auxotrophic mutant [46]. All PCR verified transformants grew slowly on MM with 2% glucose agar plates. Three independent Δ*bglR* mutants were selected for further assessment. We subsequently tested growth of the Δ*bglR* mutant in minimal medium with ammonium sulfate, MM((NH_4_)_2_SO_4_) as the sole nitrogen source with glucose, maltose, trehalose, amylose or amylopectin as the sole carbon source. In contrast to parental WT strain QM6a, almost no growth of the Δ*bglR* mutants in MM-glucose, MM-amylopectin or MM-trehalose was observed (Fig 7A). Surprisingly, neither the parental QM6a strain nor the Δ*bglR* mutant grew in MM when maltose or amylose was used as the sole carbon source.

To assess the influence of glutamine on glucose utilization in the Δ*bglR* mutant, we measured changes in glucose concentration in liquid cultures of QM6a and the Δ*bglR* mutant in MM((NH_4_)_2_SO_4_) or MM(Gln) with 2% glucose as the sole carbon source. This approach was chosen because *T*. *reesei* utilized glutamine as a carbon source more efficiently than *N*. *crassa*, which prevented an unambiguous conclusion about glucose consumption rate based on growth phenotypes (S2 Fig). In MM((NH_4_)_2_SO_4_), only 11% of the glucose in the medium was used after 2 days by the Δ*bglR* mutant versus a 90% reduction in glucose levels in the parental QM6a strain (Fig 7B). In MM(Gln), an increase of glucose consumption to 60% by the Δ*bglR* mutant was detected when glutamine was used as the nitrogen source, while QM6a showed similar glucose consumption on MM(Gln) as MM((NH_4_)_2_SO_4_) (Fig 7B). These data suggest that, like COL-26 in *N*. *crassa*, BglR also plays a critical role in regulating starch degradation and primary carbon and nitrogen metabolism in *T*. *reesei*. Based on the phylogenetic analyses, such a multi-regulatory role may also be conserved in *col-26* orthologs in many other filamentous fungal species.

## Discussion

In nature, filamentous fungi must integrate data from available carbon sources to coordinate with nitrogen, phosphorus and sulfur assimilation for optimal growth. How this coordination is achieved in these organisms is currently not clear, as most studies evaluate physiological/transcriptional differences based on comparison between single carbon or nitrogen sources. In this study, we identified a conserved regulator, COL-26, that plays a role in coordinating the utilization of starch components with nitrogen regulation.

By comparing the amylose and maltose RNA-seq data between the WT and the Δ*col-26* mutant, we identified a 363-COL-26-dependent gene set. This gene set contained many genes with functions in primary nitrogen and amino acid metabolism, including the transcription factors *vib-1*, *nit-2*, and *cpc-1*. A large percentage of these genes were also induced in WT when cells were exposed to maltose or amylose, indicating coordinate regulation of nitrogen metabolism with carbon metabolism. The down regulation of genes such as *gln-1* and *gln-2* in the Δ*col-26* mutant and its unique phenotype of poor growth on preferred carbon sources led us to speculate that an inability to coordinate carbon and nitrogen metabolism may occur in the Δ*col-26* mutant. This hypothesis was supported by the partial rescue of growth defects in the Δ*col-26* mutant by the use of glutamine as the sole nitrogen source with glucose as the carbon source. As in *N*. *crassa*, growth defects on glucose medium were noted for a *F*. *graminearum* Δ*FgART* mutant [10], a *M*. *oryzae* ΔMoCOD1 mutant [44] and a *T*. *reesei* Δ*bglR* mutant [11]. Here we provided experimental evidence that in *T*. *reesei*, BglR was essential for amylopectin and trehalose utilization and for ammonium assimilation on preferred carbon sources. Our data also showed that *T*. *reesei* cannot grow on amylose or maltose. These data indicate that, unlike *N*. *crassa*, *T*. *reesei* may rely on α-1, 6 linkages for signaling for starch degradation. Whether these functions are all conserved by the COL-26 orthologs in other filamentous fungi awaits further verification. However, as many filamentous fungi are plant pathogens of starch crops, such as *F*. *graminearum* and *M*. *oryzae*, and deletion of the *col-26* orthologs reduced pathogenicity in both these fungi, understanding the regulatory mechanisms by the COL-26 orthologs could shed light on the development of future anti-fungal strategies.

The data from metabolic analyses showed that several metabolites in the TCA cycle and GABA shunt pathway were either at a higher or lower level in the Δ*col-26* mutant as compared to WT cells. In particular, high levels of succinate and GABA persisted and succinic semialdehyde remained below detectable levels in the Δ*col-26* mutant even when glutamine was provided as the nitrogen source and growth was partially restored. The GABA shunt pathway is a metabolic route conserved among bacteria, fungi, plant and vertebrates. The role of the GABA shunt has been extensively investigated in animals and plants due to GABA being a key neurotransmitter in the central and peripheral nervous system of vertebrates and a signal molecule in response to many biotic and abiotic stresses in plants [47]. The GABA shunt in fungi has received less attention, but has been associated with nitrogen metabolism, spore germination, asexual sporulation, redox homeostasis, acidogenic growth, response to hypoxia, oxidative stress and virulence [48–53]. Besides the GABA shunt, an alternative pathway exists for GABA catabolism in many eukaryotes including *S*. *cerevisiae*, through which the intermediate metabolite, succinic semialdehyde (SSA), is reduced to γ-hydroxybutyric acid (GHB) [54]. In the Δ*col-26* mutant, it is possible that mis-regulation of enzyme activities at either the transcriptional and (or) post-transcriptional level and/or defects in the transport of glutamate or GABA between cytoplasm and the mitochondria may occur. Whether the reduction of succinic semialdehyde or the other metabolites in the mutant is caused by increased activity of enzymes in one pathway versus a re-wiring the metabolite to other pathways warrants further investigation. Our metabolite data is consistent with a regulatory role of COL-26 in the GABA shunt and in coordinating primary carbon and nitrogen metabolism for optimal fungal growth.

In addition to a role in the coordination of primary carbon and nitrogen metabolism, COL-26 is essential for the utilization of starch. In *A*. *niger*, transcriptional analyses via microarrays of carbon-limited chemostat or batch cultures growing on maltose versus xylose revealed that only three amylolytic genes *aamA* (acid α-amylase), *glaA* (glucoamylase), *agdA* (α-glucosidase) were induced by maltose [55, 56]. In *A*. *oryzae*, ten genes annotated to encode glucoamylase, maltose permease, maltase, sugar transporters and maltose O-acetyltransferase were up regulated by maltose [57]. In this study, we performed systematic transcriptional profiling of *N*. *crassa* on different components of starch, including maltose, amylose, and amylopectin. From these analyses, we identified a starch regulon consisting of 322 genes; COL-26 is required for WT expression patterns of 252 of these 322 genes. Surprisingly, our data showed that expression changes in *N*. *crassa* in response to polysaccharides of starch differed substantially from those induced by maltose (Fig 2A), where only ~1/3 of the starch regulon genes were induced (Fig 2B). Such transcriptional differences may reflect changes in signaling or utilization strategies by *Neurospora* for optimal uptake of nutrients of different forms (disaccharides versus polysaccharides, for example).

The function of a COL-26 homolog in *Aspergilli*, AmyR, the transcriptional regulator associated with maltose and starch utilization in *Aspergillus spp*., shows some divergence in function even among *Aspergillus* species. In *A*. *oryzae*, where maltose-utilizing (*MAL*) clusters are found, AmyR is important for starch degradation, but MalR is required for maltose utilization and AmyR activation [45]. In *A*. *nidulans* and *A*. *niger*, which lack *MAL* clusters, AmyR is critical for both maltose and starch utilization [5, 8]. *N*. *crassa* does not have *MAL* clusters and no protein exhibits higher homology to MalR than COL-26. Here, we demonstrated that COL-26 is essential for the utilization of maltose, amylopectin and amylose, all components of starch. Consistent with this essential role, the expression of *col-26* increased in presence of amylose, amylopectin, and under a low concentration of maltose (2 mM), while deletion of *col-26* led to decrease in expression level of 78% of the starch-regulon genes.

Genes related to cellulose degradation were among the genes that increased in expression level in the Δ*col-26* mutant. These included cellodextrin and cellobionic acid transporter genes, *cdt-2* and *cbt-1*, respectively and cellulase genes *pmo-3* and *cdh-2*. Substrates of CDT-2 and CBT-1 are in fact products from PMO-3 and CDH [38, 39]. A screen for *N*. *crassa* hypersecretors of cellulases also identified a modest increase of cellulase production in the Δ*col-26* mutant [46]. These data support the hypothesis that cellulose degradation by *N*. *crassa* is negatively regulated by a COL-26-mediated glucose repression, consistent with the robust 2-DG resistance in Δ*col-26* mutant. In support of a conserved function of COL-26, a *Penicillin oxalicum* Δ*amyR* mutant also showed decreased amylase activity and increased cellulase expression on cellulose [58]. These observations suggest an antagonizing effect between activation of amylolytic genes versus cellulase genes in filamentous fungi, which is mediated by COL-26/AmyR.

In this study, although we focused on elucidating the essential roles of COL-26 in regulating starch degradation and primary carbon and nitrogen metabolism, we also demonstrated that COL-26 and BglR were essential for trehalose utilization. Trehalose is the major internal carbohydrate reserve in *N*. *crassa* and other fungi and trehalose mobilization occurs during germination of fungal spores, a process that can be enhanced by glucose combined with a nitrogen source [59]. The *tre-1* gene, encoding trehalase, was within the starch regulon, but was not differentially expressed in the Δ*col-26* mutant on starch components. Whether the inability to utilize trehalose is a consequence of the inability of the Δ*col-26* mutant to efficiently utilize glucose (cleavage of trehalose yields two glucose molecules) is unclear. In the insect pathogen *Metarhizium acridum*, enhancing fungal utilization of trehalose, the main carbon source in insect hemolymph, has been shown to improve virulence [60]. Single *col-26* orthologs occur in the genomes of the insect-pathogenic fungi *Metarhizium acridum* and *Metarhizium robertsii*. Further study of functions of the COL-26 orthologs in trehalose utilization in these fungi may aid in developing more potent strains for insect biocontrol.

Finally, we identified a number of predicted transporter genes within the starch regulon, including *hgt-1*, *xyt-1*, NCU04963, and NCU04537, while *cdt-1* and NCU12154 were significantly induced by maltose. NCU12154 has been annotated as maltose permease based on bioinformatics analyses, although biochemical evidence is lacking. It is possible that one of these uncharacterized transporters encode a maltooligosaccharide transporter that accompanies activity of intracellular α-amylase, which are part of the starch regulon. Testing transporting activity of the predicted transporters will aid in our understanding of diverse nutrient assimilation pathways by filamentous fungi.

## Materials and methods

### Strains

*N*. *crassa Δcol-26* (FGSC 11031) and Δ*gln-1* (FGSC 19959) were obtained from the Fungal Genetics Stock Center (http://www.fgsc.net/). The P*gpd-col-26;* Δ*col-26* strain was constructed by transforming the Δ*col-26* mutant with a DNA fragment containing the *A*. *nidulans gpdA* promoter, the open reading frame and 3’ untranslated region (UTR) of *col-26*, and flanking regions homologous to the upstream and downstream genomic sequence of the *csr-1* gene. Transformants were selected for resistance for cyclosporin [61] and tested for genotypes by diagnostic PCR. The transformants with positive results were backcrossed to FGSC 2489 to obtain a *csr-1*::*PgpdA-col-26;* Δ*col-26* homokaryotic strain.

The *T*. *reesei* Δ*bglR* mutants were created by transforming protoplasts of an uridine auxotrophic strain made from QM6a (Δ*pyr-4*) [46] with two split-marker DNA fragments using method described in [62]. One of the split-marker fragment contains a ~1 kbp sequence homologous to upstream genomic sequence of the *bglR* gene followed by the promoter and the first half of the *pyr-4* coding sequence and the other contained the second half the *pyr-4* coding sequence with ~400 bp of overlap sequence with the first half of the *pyr-4* coding sequence and a ~1 kb sequence homologous to the downstream genomic sequence of the *bglR* gene. Transformants were first grown on the plates with minimal media and subsequently transferred to PDA plates for conidiation. Conidia were tested for correct integration of the *pyr-4* gene at the *bglR* locus using diagnostic PCR. The strains with the *bglR* gene disrupted were subjected to single colony purification. Three verified Δ*bglR* homokaryotic strains were used for downstream analysis.

### Culture conditions

*N*. *crassa* cultures were grown on slants, each with 3 mL of Vogel’s minimal medium (VMM) with 2% sucrose [17] and 2% agar, at 30°C in dark for 24 hours, followed by 4–10 days in constant light at 25°C to stimulate conidia production. For growth phenotype testing in 24-well plates, conidia were inoculated at 10^6^/ml into 3 mL of VMM with selected carbon and nitrogen sources in 24-well plates covered with breathable rayon film seal, and the culture were grown at 25°C in constant light with shaking at 200 rpm. The film was taken off before imaging. At least two replicates were included in each experiment and the same experiments were done at least twice.

For mycelial biomass measurement, conidia were inoculated at 10^6^/ml into 100 mL of VMM with selected carbon and nitrogen sources and grown at 25°C in constant light with shaking at 200 rpm. For crosses, one parental strain was grown on plates with synthetic crossing medium [63] for 2 weeks at room temperature for protoperithecial development. Conidia of the other parental strain were added to the plates for fertilization. Plates were kept for 3 weeks at room temperature. Ascospores were collected and activated as described [64], plated on VMM with 1% sucrose, and incubated at room temperature for 18 hrs. Germinated ascospores were transferred to VMM slants supplemented with cyclosporin or hygromycin B and screened for desired genotypes by diagnostic PCR.

*T*. *reesei* cultures were grown in either minimal media [65] for selecting transformants or with PDA for conidiation. For growth phenotype testing, conidia were inoculated at 10^6^/ml into 3 mL minimal media with a selected carbon source in 24-well plates, and the culture were grown at 28°C in dark with shaking at 200 rpm.

### Media shift experiments

For RNA-seq experiments on VMM with 2% (w/v) maltose and metabolite analyses, conidia were inoculated at 10^6^ conidia/mL into 3 mL VMM with 2% cellobiose and grown at 25°C in constant light and shaking at 200 rpm for 28 hrs. The mycelial biomass was washed twice with VMM-NC, followed by 18 hrs of incubation in VMM-NC. Mycelia were then transferred to VMM with maltose and grown 5.5 hrs for RNA-seq experiments, or transferred to VMM or VMM(Glu) with 2% (w/v) glucose and grown 5.5 hrs for metabolite profiling experiments.

For RNA-seq experiments on VMM with other carbon sources, conidia were inoculated at 10^6^ conidia/mL into 3 mL VMM with 2% sucrose and grown at 25°C in constant light and shaking at 200 rpm for 16 hrs. The mycelial biomass was washed twice with VMM-NC and then transferred to VMM with the selected carbon sources for 4 hrs prior to RNA extraction. Concentrations of carbon sources were glycerol (2 mM), fructose (2 mM), mannose (2 mM), trehalose (2 mM), sorbose (2 mM), xylose (2 mM), sucrose (2% w/v), cellobiose (2 mM), maltose (2 mM), avicel (1% w/v), amylose (1% w/v), amylopectin (1% w/v), xyloglucan (1% w/v).

### RNA-seq experiments, data processing and analyses

Mycelia of cultures were harvested by filtration and flash frozen in liquid nitrogen. RNA was extracted using the Trizol method (Invitrogen) and further purified using RNeasy kits (QIAGEN). RNA-seq libraries of WT and Δ*col-26* from 2% (w/v) maltose were prepared at the Functional Genomics Lab, a QB3-Berkeley Core Research Facility at UC Berkeley and sequenced on an Illumina HiSeq2000 at the Vincent J. Coates Genomics Sequencing Lab. Other libraries were prepared and sequenced at JGI as part of the Neurospora ENCODE CSP project. Total RNA starting material was 1 μg per sample and 10 cycles of PCR was used for library amplification. The prepared libraries were then quantified using KAPA Biosystem’s next-generation sequencing library qPCR kit and run on a Roche LightCycler 480 real-time PCR instrument. The quantified libraries were then multiplexed into pools of 9 libraries, and the pool was then prepared for sequencing on the Illumina HiSeq sequencing platform utilizing a TruSeq paired-end cluster kit, v3, and Illumina’s cBot instrument to generate a clustered flowcell for sequencing. Sequencing of the flowcell was performed on the Illumina HiSeq2000 sequencer using a TruSeq SBS sequencing kit, v3, following a 1x100 indexed run recipe.

The sequencing reads that passed filtering from the CASAVA 1.8 FASTQ files were subjected to quality score checking using the FASTX-Toolkit (http://hannonlab.cshl.edu/fastx_toolkit/). Only reads with all bases scoring greater than 22 were used to map against predicted transcripts from the *N*. *crassa* OR74A genome v12 (*Neurospora crassa* Sequencing Project, Broad Institute of Harvard and MIT http://www.broadinstitute.org/) with Tophat v2.0.4 [66]. The output bam files were sorted and indexed using the SAMtools package [67] and the indexed files were visualized in Integrative Genomics Viewer [68]. Transcript abundance reflected in FPKM was estimated with Cufflinks v2.0.2 [66] mapping against reference isoforms. Profiling data are available at the GEO (http://www.ncbi.nlm.nih.gov/geo/; Series Record GSE GSE92848 and GSE95350). For differential gene expression analysis, the bam files were first processed using the HTSeq package v0.6.0 [69] to generate raw counts, and the raw counts are subjected to differential analysis using the DESeq2 package version 1.10.1 [70].

The FungiFun2 online resource tool was used in functional enrichment analysis (https://elbe.hki-jena.de/fungifun/fungifun.php) [71]. The gene to category associations was tested for over-representation using hypergeometric distribution and the probability for false discovery rate was controlled by the Benjamini-Hochberg procedure.

### Metabolite extraction

Mycelia from 3 mL cultures in 24-well plates were harvested by filtration followed by a quick wash in distilled water. Half of biological replicates were used for metabolite extraction and the other half were dried for biomass measurement. Washed mycelia for metabolite extraction were quickly put into a tube containing 200 μL zirconia beads (0.5 mm) and 500 μL extraction buffer (80% acetonitrile, 20% water, 0.1 M formic acid) and snap frozen in liquid nitrogen. Samples were stored at -80°C until extraction before mass spectrometry (MS) analysis. For metabolite extraction, the frozen samples were immediately put in a bead-beater (BioSpec) and homogenized for 1 min, and cooled on ice. The homogenate were centrifuged at 4°C at 14 000 rpm for 5 minutes, and the supernatants were subjected to either GC-MS or LC-MS analysis.

For GC-MS analysis, 20 μL of the supernatant was collected and transferred to 1.5 mL micro-tubes containing 50 μL internal standard solution (d27-Myristic acid in methanol, 250 μM). Samples were dried under reduced pressure using a speedvac (Savant). Samples were derivatized for GC-MS analysis according to the method of Kind et al [72]. Briefly, 10 μL of methoxyamine hydrochloride dissolved in pyridine (40 mg/mL) was added to each dried sample, and shaken at 30°C at maximum speed for 90 min using a thermomixer (Eppendorf). A mixture of retention time marker standards were prepared by dissolving fatty acid methyl esters (FAMEs) of different linear chain lengths in chloroform (C8, C9, C10, C12, C14, C16 FAMES at 0.8 mg/ml, and C18, C20, C22, C24, C26, C28, C30 at 0.4 mg/ml). The FAME mixture (20 μL) was added to 1 mL of N-methyl-N-trimethylsilytrifluoroacetamide (MSTFA) containing 1% trimethylchlorosilane (TMCS), and 90 μL of the FAMEs/MSTFA solution was added to each sample. Samples were shaken at 37°C at maximum speed in a thermomixer for 30 min, and then transferred to and sealed in amber GC-MS sample vials containing glass inserts (Agilent). Extraction blanks were prepared following the above procedure but starting with empty Eppendorf tubes.

For LC-MS analysis, supernatant (350 μL) was collected and filtered through 0.2 μm spin filters (Pall) by centrifugation for 1 min at 14000 rpm. Fifty μL of the filtrate was transferred to HPLC vials containing 50 μL of an internal standard mixture solution. Samples were kept at 4°C in the LC-MS autosampler chamber. Extraction blanks were prepared in triplicate by following the above sample preparation procedure with empty microtubes.

### Metabolite profiling, data acquisition and analyses

For GC-MS analysis, samples were analyzed using an Agilent 7890 gas chromatograph (Agilent Technologies, Santa Clara, CA) connected to an Agilent 5977 single quadrupole mass spectrometer, all controlled by Agilent GC-MS MassHunter Acquisition software. Samples were injected using a Gerstel automatic liner exchange MPS system (Gerstel, Muehlheim, Germany) controlled by Maestro software. Sample injection volume was 2 μL, and the injector was operated in splitless mode. Samples were injected into the 50°C injector port which was ramped to 270°C in a 12°C/s thermal gradient and held for 3 min. The gas chromatograph was fitted with a 30m long, 0.25mm ID Rtx5Sil-MS column (Restek, Bellefonte, PA), 0.25 mm 5% diphenyl film with a 10 m integrated guard column. Initial oven temperature was set at 50°C, and the over program was as follows: ramp at 5°C/min to 65°C, held for 0.2 min; ramp at 15°C/ min to 80°C, held for 0.2 min; ramp at 15°C/min to 310°C, hold for 12 min. The mass spectrometer transfer line and ion source temperature was 250°C and 230°C, respectively. Electron ionization was at 70 eV and mass spectra were acquired from 50 to 700 m/z at 8 spectra per second. Raw data was visually inspected using Agilent MassHunter Qualitative Analysis software (Agilent Technologies, Santa Clara, CA). Agilent MassHunter Unknowns Analysis software v. B.07.00 (Agilent Technologies, Santa Clara, CA) was used to perform peak deconvolution and library matching. A library match score was calculated for using FAME markers for retention time calibration, and matching mass fragmentation spectra to those in the Fiehn GC-MS Metabolomics RTL Library [72]. Metabolites of interest were only included in further analysis if their library match scores were greater than 75%. The identities of some metabolites with scores lower than 90% were confirmed by comparing mass spectra and retention times with that of authentic reference standards (S6 Table). Mass spectral and retention time data from identified target metabolites were used to make an analysis method in MassHunter Quantitative Analysis Software for GCMS (v.B.07.00). For each metabolite, a quantifier ion and two qualifier ions were defined to produce an extracted ion chromatogram in a specified retention time window. Integration of the extracted ion chromatogram peaks yielded peak areas that were further normalized by the mean of dry fungal biomass from biological replicates. The normalized peak areas were used for comparing the relative abundance of metabolites across samples.

Targeted LC-MS analysis was performed for select metabolites not detected by GC-MS (S7 Table). Samples were analyzed on an Agilent 6550 ESI-QTOF LCMS fitted with a Merck SeQuant Zic-HILIC column (150 x 1 mm, 3.5 mm, 100 Å) with a guard column. Mobile phase consisted of 5% ammonium acetate in water (solvent A), and 5% ammonium acetate in water-acetonitrile (10:90) (solvent B). The following LC solvent time-table was used: 0 min, 100% B; 1.5 min, 100% B; 25 min, 50% B; 26 min, 35% B; 32 min, 35% B; 33 min, 100% B; 40 min, 100% B. Flow rate: 0.25 ml/min; injection volume: 2 μL. Each sample was analyzed in positive and negative ionization mode. Raw data was analyzed using Agilent MassHunter Qualitative analysis. Extracted ion chromatograms were produced from raw scan data using calculated m/z values for target metabolites, corresponding to their molecular ion and potential adducts: (M+H)^+^, (M+Na)^+^, (M+K)^+^ for Positive mode; (M-H)^-^, (M+COOH)^-^, (M+CH3COOH)^-^ for Negative mode. The identity of detected ions were confirmed by comparing retention time with reference standards, or checked by performing MS/MS analysis of the target ion, and comparing ion fragments with those in the METLIN online database. Integration of the extracted ion chromatogram peaks yielded peak areas that were further normalized by the mean of dry fungal biomass from biological replicates. Normalized peak areas were used for comparing the relative abundance of target metabolites across samples.

For differential metabolite analysis, the normalized peak areas were log transformed and then used in the independent t-test of hypothesis that there is no difference between WT and the mutant. P values of less than 0.05 were considered significantly different and values between 0.05 and 0.1 were interpreted as indicating a trend toward statistical significance. Four biological replicates measured by GC-MS and two biological replicates measured by LC-MS were used in differential analysis. All metabolites that were found to be either significantly different or with a trend toward statistical significance were subjected to hierarchical clustering analysis.

Hierarchical clustering analysis is performed with Cluster 3.0 [73] using log transformed mean of normalized peak areas from biological replicates. The values were centered to the mean across different growth conditions and normalized on a per metabolite basis. Average linkage clustering was performed with Euclidean distance as the similarity metric.

### Phylogenetic analysis of putative *col-26* orthologs

Protein sequences of selected ascomycetes were downloaded from JGI Mycosm [74] and used to construct a local protein database using the NCBI BLAST+ application (version 2.2.31) (S5 Table). The putative COL26 orthologs were searched in the database using BLASTP with a cut-off E value less than e-20. All hits were tested by reciprocally BLASTP against the *N*. *crassa* database and only ones that resulted in COL26 as the best hit were retained for protein sequence alignment. Protein sequences of the putative COL26 orthologs from selected species were aligned using three different programs: Clustal Omega [75], MAFFT [76], and MUSCLE [77], and the best alignment was chosen and further trimmed using trimAl [78]. The trimmed alignment file was used for phylogenetic tree construction by the RAxML program with 200 bootstraps [79]. The result were visualized and edited in iTOL (http://itol.embl.des/) [80].

## Supporting information

S1 Table Expression data from WT cells. Sheet 1: Genes with statistically significant expression changes in WT cells when exposed to amylose or amylopectin relative to WT cells exposed to no carbon and sucrose. Sheet 2: Genes with statistically significant expression changes in WT cells when exposed to maltose relative to WT cells exposed to no carbon and sucrose. Sheet 3: Overlap between maltose-inducible gene set and starch regulon in WT cells.
(XLSX)

S2 Table Expression data from *Δcol-26* cells. Sheet 1: Genes with significant expression changes under amylose conditions in the Δ*col-26* mutant relative to WT. Sheet 2: Genes with significant expression changes under maltose conditions in the Δ*col-26* mutant relative to WT.
(XLSX)

S3 Table List of genes differentially expressed in Δ*col-26* mutant under both maltose and amylose conditions (the *col-26-*dependent and the *col-26*-reduced gene sets). (XLSX)

S4 Table Intracellular metabolites identified by either GC or LC-MS in the Δ*col-26* or Δ*gln-1* mutants versus WT cells. (XLS)

S5 Table List of the *col-26* homologs identified in the genomes of 44 ascomycete species. (XLSX)

S6 Table Metabolites identified by GC-MS using the Fiehn metabolite library, and corresponding ions used as quantifier and qualifier ions for extracting peaks from raw data. (PDF)

S7 Table Metabolites identified by LC-MS and corresponding ions used to extract peaks from raw data for relative quantitation. (PDF)

S1 Fig Growth of WT, Δ*col-26* mutants, and Δ*gln-1* mutant on VMM with an amino acid (2% w/v) as both carbon and nitrogen sources and on VMM (NH_4_NO_3_) with an amino acid (2% w/v) as the carbon source. (PDF)

S2 Fig Growth of WT, Δ*bglR* mutants on MM with glutamine or ammonium sulfate as the nitrogen source and either glutamine or sugar as the carbon source. (PDF)

## References

[1] Tsukagoshi N, Kobayashi T, Kato M (2001) Regulation of the amylolytic and (hemi)cellulolytic genes in aspergilli. J Gen Appl Microbiol 47:1–19. 12483563 10.2323/jgam.47.1

[2] Vu VV, Beeson WT, Span EA, Farquhar ER, Marletta MA (2014) A family of starch-active polysaccharide monooxygenases. Proc Natl Acad Sci U S A 111:13822–7. 10.1073/pnas.1408090111 25201969 PMC4183312

[3] Lachmund A, Urmann U, Minol K, Wirsel S, Ruttkowski E (1993) Regulation of alpha-amylase formation in *Aspergillus oryzae* and *Aspergillus nidulans* transformants. Curr Microbiol 26:47–51.

[4] Morkeberg R, Carlsen M, Nielsen J (1995) Induction and repression of alpha-amylase production in batch and continuous cultures of *Aspergillus oryzae*. Microbiol 141:2449–54. 10.1099/13500872-141-10-2449 7582005

[5] vanKuyk PA, Benen JA, Wosten HA, Visser J, de Vries RP (2012) A broader role for AmyR in *Aspergillus niger*: regulation of the utilisation of D-glucose or D-galactose containing oligo- and polysaccharides. Appl Microbiol Biotechnol 93:285–93. 10.1007/s00253-011-3550-6 21874276 PMC3251782

[6] Petersen KL, Lehmbeck J, Christensen T (1999) A new transcriptional activator for amylase genes in *Aspergillus*. Mol Gen Genet 262:668–76. 10628849 10.1007/s004380051129

[7] Gomi K, Akeno T, Minetoki T, Ozeki K, Kumagai C, Okazaki N, et al (2000) Molecular cloning and characterization of a transcriptional activator gene, *amyR*, involved in the amylolytic gene expression in *Aspergillus oryzae*. Biosci Biotechnol Biochem 64:816–27. 10.1271/bbb.64.816 10830498

[8] Tani S, Katsuyama Y, Hayashi T, Suzuki H, Kato M, Gomi K, et al (2001) Characterization of the *amyR* gene encoding a transcriptional activator for the amylase genes in *Aspergillus nidulans*. Curr Genet 39:10–5. 11318101 10.1007/s002940000175

[9] Liu G, Zhang L, Qin Y, Zou G, Li Z, Yan X, et al (2013) Long-term strain improvements accumulate mutations in regulatory elements responsible for hyper-production of cellulolytic enzymes. Sci Rep 3:1569 10.1038/srep01569 23535838 PMC3610096

[10] Oh M, Son H, Choi GJ, Lee C, Kim JC, Kim H, et al (2016) Transcription factor ART1 mediates starch hydrolysis and mycotoxin production in *Fusarium graminearum* and *F*. *verticillioides*. Mol Plant Pathol 17:755–68. 10.1111/mpp.12328 26456718 PMC6638531

[11] Nitta M, Furukawa T, Shida Y, Mori K, Kuhara S, Morikawa Y, et al (2012) A new Zn(II)(2)Cys(6)-type transcription factor BglR regulates beta-glucosidase expression in *Trichoderma reesei*. Fungal Genet Biol 49:388–97. 10.1016/j.fgb.2012.02.009 22425594

[12] Le Crom S, Schackwitz W, Pennacchio L, Magnuson JK, Culley DE, Collett JR, et al (2009) Tracking the roots of cellulase hyperproduction by the fungus *Trichoderma reesei* using massively parallel DNA sequencing. Proc Natl Acad Sci U S A. 106:16151–6. 10.1073/pnas.0905848106 19805272 PMC2752593

[13] Colot HV, Park G, Turner GE, Ringelberg C, Crew CM, Litvinkova L, et al (2006) A high-throughput gene knockout procedure for *Neurospora* reveals functions for multiple transcription factors. Proc Natl Acad Sci U S A 103:10352–7. 10.1073/pnas.0601456103 16801547 PMC1482798

[14] Xiong Y, Sun JP, Glass NL (2014) VIB1, a link between glucose signaling and carbon catabolite repression, is essential for plant cell wall degradation by *Neurospora crassa*. PLoS Genet 10:e1004500 10.1371/journal.pgen.1004500 25144221 PMC4140635

[15] Payen A (1843) (Rapporteur) Extrait d'un rapport adresse a M. Le Marechal Duc de Dalmatie, Ministre de la Guerre, President du Conseil, sur une alteration extraordinaire du pain de munition. Annales de Chimie et de Physique 9:18.

[16] Perkins DD, Radford A, Sachs MS (2000) The Neurospora Compendium: Chromosomal Loci. Academic Press; 325 p.

[17] Vogel H (1956) A convenient growth medium for *Neurospora* (medium N). Microb Genet Bull 13:2–43.

[18] Lombard V, Golaconda Ramulu H, Drula E, Coutinho PM, Henrissat B (2014) The carbohydrate-active enzymes database (CAZy) in 2013. Nucleic Acids Res 42:D490–5. 10.1093/nar/gkt1178 24270786 PMC3965031

[19] Xie X, Wilkinson HH, Correa A, Lewis ZA, Bell-Pedersen D, Ebbole DJ (2004) Transcriptional response to glucose starvation and functional analysis of a glucose transporter of *Neurospora crassa*. Fungal Genet Biol 41:1104–19. 10.1016/j.fgb.2004.08.009 15531214

[20] Li J, Lin L, Li H, Tian C, Ma Y (2014) Transcriptional comparison of the filamentous fungus *Neurospora crassa* growing on three major monosaccharides D-glucose, D-xylose and L-arabinose. Biotechnol Biofuels 7:31 10.1186/1754-6834-7-31 24581151 PMC4015282

[21] Park G, Ryu YH, Hong YJ, Choi EH, Uhm HS (2012) Cellular and molecular responses of *Neurospora crassa* to non-thermal plasma at atmospheric pressure. Appl Phys Lett 100:063703.

[22] Montenegro-Montero A, Goity A, Larrondo LF (2015) The bZIP transcription factor HAC-1 is involved in the unfolded protein response and is necessary for growth on cellulose in *Neurospora crassa*. PloS One 10:e0131415 10.1371/journal.pone.0131415 26132395 PMC4488935

[23] Coradetti ST, Craig JP, Xiong Y, Shock T, Tian C, Glass NL (2012) Conserved and essential transcription factors for cellulase gene expression in ascomycete fungi. Proc Natl Acad Sci U S A 109:7397–402. 10.1073/pnas.1200785109 22532664 PMC3358856

[24] Craig JP, Coradetti ST, Starr TL, Glass NL (2015) Direct target network of the *Neurospora crassa* plant cell wall deconstruction regulators CLR-1, CLR-2, and XLR-1. MBio 6:e01452–15. 10.1128/mBio.01452-15 26463163 PMC4620465

[25] Coradetti ST, Xiong Y, Glass NL (2013) Analysis of a conserved cellulase transcriptional regulator reveals inducer-independent production of cellulolytic enzymes in *Neurospora crassa*. MicrobiologyOpen. 2:595–609 10.1002/mbo3.94 23766336 PMC3948607

[26] Ruepp A, Zollner A, Maier D, Albermann K, Hani J, Mokrejs M, et al (2004) The FunCat, a functional annotation scheme for systematic classification of proteins from whole genomes. Nucleic Acids Res 32:5539–45. 10.1093/nar/gkh894 15486203 PMC524302

[27] Dementhon K, Iyer G, Glass NL (2006) VIB-1 is required for expression of genes necessary for programmed cell death in *Neurospora crassa*. Eukaryot Cell 5:2161–73. 10.1128/EC.00253-06 17012538 PMC1694810

[28] Paluh JL, Orbach MJ, Legerton TL, Yanofsky C (1988) The cross-pathway control gene of *Neurospora crassa*, *cpc-1*, encodes a protein similar to GCN4 of yeast and the DNA-binding domain of the oncogene v-jun-encoded protein. Proc Natl Acad Sci U S A 85:3728–32. 2967496 10.1073/pnas.85.11.3728 PMC280291

[29] Barthelmess IB (1982) Mutants affecting amino acid cross-pathway control in *Neurospora crassa*. Genet Res 39:169–85. 6211391 10.1017/s0016672300020863

[30] Tian C, Kasuga T, Sachs MS, Glass NL(2007) Transcriptional profiling of cross pathway control in *Neurospora crassa* and comparative analysis of the Gcn4 and CPC1 regulons. Eukaryot Cell 6:1018–29. 10.1128/EC.00078-07 17449655 PMC1951524

[31] Marzluf GA (1981) Regulation of nitrogen metabolism and gene expression in fungi. Microbiol Rev 45:437–61. 6117784 10.1128/mr.45.3.437-461.1981 PMC281519

[32] Magill JM, Edwards ES, Sabina RL, Magill CW (1976) Depression of uracil uptake by ammonium in *Neurospora crassa*. J Bacteriol 127:1265–9. 134026 10.1128/jb.127.3.1265-1269.1976 PMC232919

[33] Galazka JM, Tian C, Beeson WT, Martinez B, Glass NL, Cate JH (2010) Cellodextrin transport in yeast for improved biofuel production. Science 330:84–6. 10.1126/science.1192838 20829451

[34] Cai P, Gu R, Wang B, Li J, Wan L, Tian C, et al (2014) Evidence of a critical role for cellodextrin transporter 2 (CDT-2) in both cellulose and hemicellulose degradation and utilization in *Neurospora crassa*. PloS One 9:e89330 10.1371/journal.pone.0089330 24586693 PMC3930720

[35] Li X, Yu VY, Lin Y, Chomvong K, Estrela R, Park A, et al (2015) Expanding xylose metabolism in yeast for plant cell wall conversion to biofuels. Elife 4. 10.7554/eLife.05896 PMC4338637 25647728

[36] Xiong Y, Coradetti ST, Li X, Gritsenko MA, Clauss T, Petyuk V, et al (2014) The proteome and phosphoproteome of *Neurospora crassa* in response to cellulose, sucrose and carbon starvation. Fungal Genet Biol 72:21–33. 10.1016/j.fgb.2014.05.005 24881580 PMC4247816

[37] Li X, Chomvong K, Yu VY, Liang JM, Lin Y, Cate JH (2015) Cellobionic acid utilization: from *Neurospora crassa* to *Saccharomyces cerevisiae*. Biotechnol Biofuels 8:120 10.1186/s13068-015-0303-2 26279678 PMC4537572

[38] Phillips CM, Beeson WT, Cate JH, Marletta MA (2011) Cellobiose dehydrogenase and a copper-dependent polysaccharide monooxygenase potentiate cellulose degradation by *Neurospora crassa*. ACS Chem Biol 6:1399–406. 10.1021/cb200351y 22004347

[39] Li X, Beeson WTt, Phillips CM, Marletta MA, Cate JH (2012) Structural basis for substrate targeting and catalysis by fungal polysaccharide monooxygenases. Structure 20:1051–61. 10.1016/j.str.2012.04.002 22578542 PMC3753108

[40] Dunn-Coleman NS, Garrett RH (1980) The role for glutamine synthetase and glutamine metabolism in nitrogen metabolite repression, a regulatory phenomenon in the lower eukaryote *Neurospora crassa*. Mol Gen Genet 179:25–32. 6109228 10.1007/BF00268442

[41] Metzenberg RL (2004) Bird Medium: an alternative to Vogel Medium. Fungal Genet Newsl 51:19–20.

[42] Madi L, McBride SA, Bailey LA, Ebbole DJ (1997) *rco-3*, a gene involved in glucose transport and conidiation in *Neurospora crassa*. Genetics 146:499–508. 9178001 10.1093/genetics/146.2.499 PMC1207992

[43] Brown NA, de Gouvea PF, Krohn NG, Savoldi M, Goldman GH (2013) Functional characterisation of the non-essential protein kinases and phosphatases regulating *Aspergillus nidulans* hydrolytic enzyme production. Biotechnol Biofuels 6:91 10.1186/1754-6834-6-91 23800192 PMC3698209

[44] Chung H, Choi J, Park SY, Jeon J, Lee YH (2013) Two conidiation-related Zn(II)2Cys6 transcription factor genes in the rice blast fungus. Fungal Genet Biol 61:133–41 10.1016/j.fgb.2013.10.004 24140150

[45] Hasegawa S, Takizawa M, Suyama H, Shintani T, Gomi K (2010) Characterization and expression analysis of a maltose-utilizing (MAL) cluster in *Aspergillus oryzae*. Fungal Genet Biol 47:1–9. 10.1016/j.fgb.2009.10.005 19850146

[46] Reilly MC, Qin L, Craig JP, Starr TL, Glass NL (2015) Deletion of homologs of the SREBP pathway results in hyper-production of cellulases in *Neurospora crassa* and *Trichoderma reesei*. Biotechnol Biofuels 8:121 10.1186/s13068-015-0297-9 26288653 PMC4539670

[47] Michaeli S, Fromm H (2015) Closing the loop on the GABA shunt in plants: are GABA metabolism and signaling entwined? Front Plant Sci 6:419 10.3389/fpls.2015.00419 26106401 PMC4460296

[48] Guo M, Chen Y, Du Y, Dong Y, Guo W, Zhai S, et al (2011) The bZIP transcription factor MoAP1 mediates the oxidative stress response and is critical for pathogenicity of the rice blast fungus *Magnaporthe oryzae*. PLoS Pathog 7:e1001302 10.1371/journal.ppat.1001302 21383978 PMC3044703

[49] Mead O, Thynne E, Winterberg B, Solomon PS (2013) Characterising the role of GABA and its metabolism in the wheat pathogen *Stagonospora nodorum*. PloS One. 8:e78368 10.1371/journal.pone.0078368 24265684 PMC3827059

[50] Kumar S, Punekar NS, SatyaNarayan V, Venkatesh KV (2000) Metabolic fate of glutamate and evaluation of flux through the 4-aminobutyrate (GABA) shunt in *Aspergillus niger*. Biotechnol Bioeng 67:575–84. 10649232

[51] Solomon PS, Oliver RP (2002) Evidence that gamma-aminobutyric acid is a major nitrogen source during *Cladosporium fulvum* infection of tomato. Planta 214:414–20. 11855646 10.1007/s004250100632

[52] Kumar S, Punekar NS (1997) The metabolism of 4-aminobutyrate (GABA) in fungi. Mycol Res 101:403–9.

[53] Masuo S, Terabayashi Y, Shimizu M, Fujii T, Kitazume T, Takaya N (2010) Global gene expression analysis of *Aspergillus nidulans* reveals metabolic shift and transcription suppression under hypoxia. Mol Genet Genomics 284:415–24. 10.1007/s00438-010-0576-x 20878186

[54] Bach B, Meudec E, Lepoutre JP, Rossignol T, Blondin B, Dequin S, et al (2009) New insights into {gamma}-aminobutyric acid catabolism: Evidence for {gamma}-hydroxybutyric acid and polyhydroxybutyrate synthesis in *Saccharomyces cerevisiae*. Appl Environ Microbiol 75:4231–9. 10.1128/AEM.00051-09 19411412 PMC2704823

[55] Yuan XL, van der Kaaij RM, van den Hondel CA, Punt PJ, van der Maarel MJ, Dijkhuizen L, et al (2008) *Aspergillus niger* genome-wide analysis reveals a large number of novel alpha-glucan acting enzymes with unexpected expression profiles. Mol Genet Genomics 279:545–61. 10.1007/s00438-008-0332-7 18320228 PMC2413074

[56] Jorgensen TR, Goosen T, Hondel CA, Ram AF, Iversen JJ (2009) Transcriptomic comparison of *Aspergillus niger* growing on two different sugars reveals coordinated regulation of the secretory pathway. BMC Genomics 10:44 10.1186/1471-2164-10-44 19166577 PMC2639373

[57] Vongsangnak W, Salazar M, Hansen K, Nielsen J (2009) Genome-wide analysis of maltose utilization and regulation in aspergilli. Microbiol 155:3893–902. 10.1099/mic.0.031104-0 19696104

[58] Li Z, Yao G, Wu R, Gao L, Kan Q, Liu M, et al (2015) Synergistic and dose-controlled regulation of cellulase gene expression in *Penicillium oxalicum*. PLoS Genet 11:e1005509 10.1371/journal.pgen.1005509 26360497 PMC4567317

[59] Thevelein JM. Regulation of trehalose mobilization in fungi (1984) Microbiol Rev 48:42–59. 6325857 10.1128/mr.48.1.42-59.1984 PMC373002

[60] Peng G, Jin K, Liu Y, Xia Y (2015) Enhancing the utilization of host trehalose by fungal trehalase improves the virulence of fungal insecticide. Appl Microbiol Biotechnol 99:8611–8. 10.1007/s00253-015-6767-y 26115754

[61] Bardiya N, Shiu PK (2007) Cyclosporin A-resistance based gene placement system for *Neurospora crassa*. Fungal Genet Biol 44:307–14. 10.1016/j.fgb.2006.12.011 17320431

[62] Catlett NL, Yoder OC, Turgeon BG (2003) Split-marker recombination for efficient targeted deletion of fungal genes. Fungal Genet Newsl 50:9–11.

[63] Westergaard M, Mitchell HK (1947) Neurospora V. A synthetic medium favoring sexual reproduction. Am J Botany 34:573–7.

[64] Davis RH, Serres FJD (1970) Genetic and microbiological research techniques for *Neurospora crassa*. Meth Enzymol 17:79–143.

[65] Penttila M, Nevalainen H, Ratto M, Salminen E, Knowles J (1987) A versatile transformation system for the cellulolytic filamentous fungus *Trichoderma reesei*. Gene 61:155–64. 3127274 10.1016/0378-1119(87)90110-7

[66] Trapnell C, Roberts A, Goff L, Pertea G, Kim D, Kelley DR, et al (2012) Differential gene and transcript expression analysis of RNA-seq experiments with TopHat and Cufflinks. Nat Protoc 7:562–78. 10.1038/nprot.2012.016 22383036 PMC3334321

[67] Li H, Handsaker B, Wysoker A, Fennell T, Ruan J, Homer N, et al (2009) The sequence alignment/map format and SAMtools. Bioinformatics 25:2078–9. 10.1093/bioinformatics/btp352 19505943 PMC2723002

[68] Thorvaldsdottir H, Robinson JT, Mesirov JP (2013) Integrative Genomics Viewer (IGV): high-performance genomics data visualization and exploration. Brief Bioinform 14:178–92. 10.1093/bib/bbs017 22517427 PMC3603213

[69] Anders S, Pyl PT, Huber W (2015) HTSeq—a Python framework to work with high-throughput sequencing data. Bioinformatics 31:166–9. 10.1093/bioinformatics/btu638 25260700 PMC4287950

[70] Love MI, Huber W, Anders S (2014) Moderated estimation of fold change and dispersion for RNA-seq data with DESeq2. Genome Biol 15:550 10.1186/s13059-014-0550-8 25516281 PMC4302049

[71] Priebe S, Kreisel C, Horn F, Guthke R, Linde J (2015) FungiFun2: a comprehensive online resource for systematic analysis of gene lists from fungal species. Bioinformatics 31:445–6. 10.1093/bioinformatics/btu627 25294921 PMC4308660

[72] Kind T, Wohlgemuth G, Lee DY, Lu Y, Palazoglu M, Shahbaz S, et al (2009) FiehnLib: mass spectral and retention index libraries for metabolomics based on quadrupole and time-of-flight gas chromatography/mass spectrometry. Anal Chem 81:10038–48. 10.1021/ac9019522 19928838 PMC2805091

[73] de Hoon MJ, Imoto S, Nolan J, Miyano S (2004) Open source clustering software. Bioinformatics 20:1453–4. 10.1093/bioinformatics/bth078 14871861

[74] Grigoriev IV, Nikitin R, Haridas S, Kuo A, Ohm R, Otillar R, et al (2014) MycoCosm portal: gearing up for 1000 fungal genomes. Nucleic Acids Res 42:D699–704. 10.1093/nar/gkt1183 24297253 PMC3965089

[75] Sievers F, Wilm A, Dineen D, Gibson TJ, Karplus K, Li W, et al (2011) Fast, scalable generation of high-quality protein multiple sequence alignments using Clustal Omega. Mol Syst Biol 7:539 10.1038/msb.2011.75 21988835 PMC3261699

[76] Katoh K, Standley DM (2013) MAFFT multiple sequence alignment software version 7: improvements in performance and usability. Mol Biol Evol 30:772–80. 10.1093/molbev/mst010 23329690 PMC3603318

[77] Edgar RC (2004) MUSCLE: multiple sequence alignment with high accuracy and high throughput. Nucleic Acids Res 32:1792–7. 10.1093/nar/gkh340 15034147 PMC390337

[78] Capella-Gutierrez S, Silla-Martinez JM, Gabaldon T (2009) trimAl: a tool for automated alignment trimming in large-scale phylogenetic analyses. Bioinformatics 25:1972–3. 10.1093/bioinformatics/btp348 19505945 PMC2712344

[79] Stamatakis A (2006) RAxML-VI-HPC: maximum likelihood-based phylogenetic analyses with thousands of taxa and mixed models. Bioinformatics 22:2688–90. 10.1093/bioinformatics/btl446 16928733

[80] Letunic I, Bork P (2016) Interactive tree of life (iTOL) v3: an online tool for the display and annotation of phylogenetic and other trees. Nucleic Acids Res 44:W242–5. 10.1093/nar/gkw290 27095192 PMC4987883

