# Supplementary material for: A fungal transcription factor essential for starch degradation affects integration of carbon and nitrogen metabolism
Source: PLoS Genet. 2017 May 3;13(5):e1006737. doi: 10.1371/journal.pgen.1006737 (PMC5435353; doi:10.1371/journal.pgen.1006737)

Table S6. Metabolites identified by GC-MS using the Fiehn Metabolite Library, and corresponding ions used as quantifier and qualifier ions for extracting peaks from raw data.

Metabolite	Name in Fiehn database (*NIST database)	RT library (min)	RT (min)	Quantifier ion	Qualifier ions (% relative response)
L-leucine	[6106] L-leucine 1 [8.298]	8.65	8.70	86	75.0 (15.0), 73.0 (12.0)
L-Isoleucine	[791] DL-isoleucine 1 [8.576]	8.91	8.98	86	75.0 (20.0), 69.0 (20.0)
L-proline	[145742] L-proline 1 [8.567]	8.91	9.01	70	70.1 (100.0), 103.0 (8.0)
L-valine	[6287] L-valine 2 [9.151]	9.46	9.38	144.1	73.0 (29.0), 218.0 (20.0)
urea[a]	[1176] urea [9.599]	9.81	9.77	189.1	171.1 (25.7), 190.1 (18.0)
L-serine	[5951] L-serine 1 [9.706]	9.91	9.95	132.1	116.1 (79.7), 73.0 (56.9)
L-threonine	[6288] L-threonine 1 [10.224]	10.44	10.43	73	117.1 (99.5), 73.1 (91.8)
glycine	[750] glycine [10.456]	10.68	10.59	174.1	175.1 (25.3), 147.0 (23.0)
succinic acid[a]	[1110] succinic acid [10.509]	10.73	10.69	147	147.1 (100), 73.1 (36.6)
fumaric acid[a]	[444972] fumaric acid [10.94]	10.91	11.16	245.1	246.1 (20.2), 73.0 (42.4)
L-homoserine	[12647] L-homoserine 1 [11.141]	12.46	11.33	164.1	103.1 (57.0), 73.0 (48.9)
L-alanine	[5950] L-alanine 2 [11.182]	11.18	11.31	188.1	100.0 (24.1), 73.0 (20.8)
L-methionine	[6137] L-methionine 1 [11.835]	11.89	12.08	104.1	56.0 (69.4), 130.1 (61.4)
D-malic acid	[92824] D-malic acid [12.794]	12.93	12.90	147.1	73.1 (99.9), 147.0 (99.5)
aspartic acid	[5960] aspartic acid 2 [13.207]	13.38	13.32	160.1	73.0 (67.6), 73.1 (62.5)
L-glutamic acid	[33032] L-glutamic acid 3 [13.232]	13.41	13.36	156.1	73.0 (40.7), 258.1 (7.5)
gamma-aminobutyric acid (GABA) [a]	[119] gamma-aminobutyric acid (GABA) [13.326]	13.25	13.48	174.1	147.0 (29.4), 73.0 (29.4)
L-cysteine[a]	[594] L-cysteine 2 [13.582]	13.55	13.76	220	218.0 (100.0), 100.0 (22.0)
phenylalanine	[994] Phenylalanine 1 [13.545]	13.51	13.82	120	120.1 (100.0), 146.1 (50.1)
alpha-ketoglutarate[a]	[51] alpha ketoglutaric acid [13.859]	13.85	13.97	73	73.1 (100.0), 198.0 (30.3)
Phosphoenolpyruvate[a]	[1005] phosphoenolpyruvic acid [14.176]	14.20	14.22	369	73.0 (152.9), 211.0 (87.0)
putrescine	[1045] putrescine [15.709]	15.68	15.87	174.1	175.1 (19.6), 230.1 (19.4)
L-glutamine	[738] L-glutamine 3 [16.092]	16.11	16.25	156.1	73.0 (69.2), 73.1 (64.4)
citric acid[a]	[311] citric acid [16.615]	16.68	16.70	273.1	273.2 (92.1), 347.1 (20.5)
L-ornithine	[6262] L-ornithine 2 [16.632]	16.70	16.75	142.1	174.1 (75.2), 73.1 (50.6)
citrulline	[9750] citrulline 2 [16.691]	16.76	16.79	157.1	256.1 (81.1), 73.1 (37.4)
D-glucose[a]	[24749] D-glucose 1 [17.426]	17.57	17.53	319.4	205.2 (85.3), 73.0 (81.5)
L-histidine	[6274] L-histidine 3 [17.658]	17.82	17.80	154.1	174.1 (44.2), 254.2 (35.1)
L-lysine	[5962] L-lysine 2 [17.643]	17.81	17.88	174.1	317.3 (68.0), 317.1 (62.8)
tyrosine	[6057] tyrosine 2 [17.871]	17.88	18.11	218.1	280.0 (15.0), 219.2 (22.3)
palmitic acid[a]	[985] palmitic acid [18.846]	18.94	19.13	312.7	313.4 (95.7), 313.3 (94.7)
allantoin[a]	[204] allantoin 3 [19.167]	19.29	19.27	259.1	189.1 (62.7), 100.0 (25.4)
uric acid	[1175] uric acid 1 [19.331]	19.47	19.55	441.2	456.2 (81.3), 442.2 (39.8)
L-tryptophan	[6305] L-Tryptophan 2 [20.446]	20.55	20.65	218.1	218.2 (93.8), 130.0 (78.0)
linoleic acid[a]	[5280450] linoleic acid [20.399]	20.48	20.66	337.3	262.3 (48.8), 117.1 (32.6)
oleic acid[a]	[445639] oleic acid [20.504]	20.60	20.71	339.3	202.1 (649.3), 145.0 (92.2)
stearic acid[a]	[5281] stearic acid [20.675]	20.78	20.92	341.4	132.1 (76.8), 201.1 (14.3)
fructose-6-phosphate*	d-Fructose, 1,3,4,5-tetrakis-O-(trimethylsilyl)-,o-methyloxime, 6-[bis(trimethylsilyl) phosphate]	n/a	21.33	387.2	357.1 (41.4), 160.1 (30.8)
D-glucose-6-phosphate[a]	[439958] D-glucose-6-phosphate 1 [21.394]	21.61	21.44	387.1	299.1 (199.5), 217.1 (54.8)
trehalose[a]	[7427] D-(+) trehalose [24.752]	24.96	24.79	361.2	191.2 (68.4), 217.1 (27.9
squalene	[5280370] squalene [25.251]	25.58	25.46	69	69.1 (100.0), 81.1 (64.0)
ergosterol[a]	[444679] ergosterol [28.09]	28.41	28.88	363.3	363.4 (96.2), 337.3 (68.4)

*Metabolite identified with the aid of NIST metabolite library.
[a]Metabolite identity confirmed with authentic standard.

Mass fragmentation spectra of identified metabolites in metabolite database compared to mass spectrum in representative sample.

L-Leucine

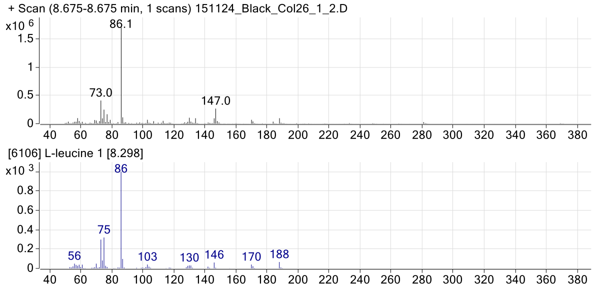

L-Isoleucine

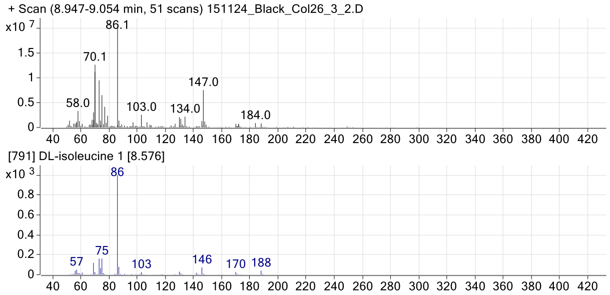

L-Proline

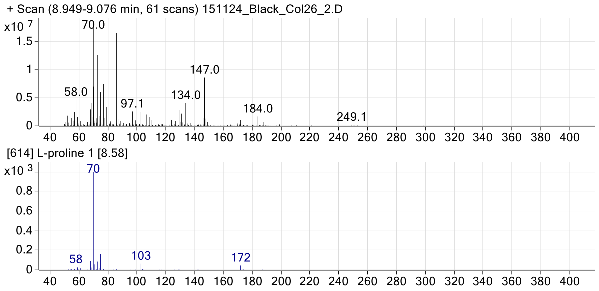

L-Valine

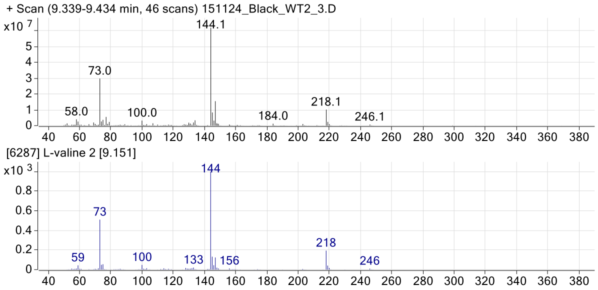

Urea

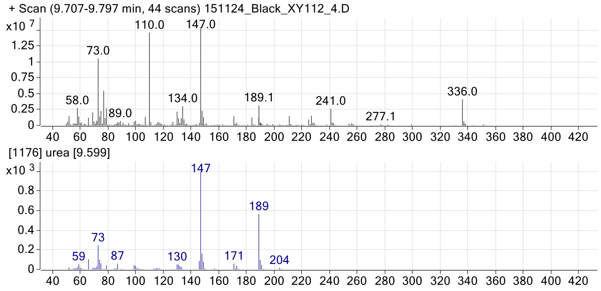

L-Serine

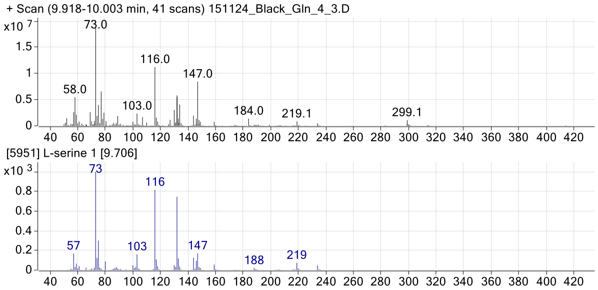

L-Threonine

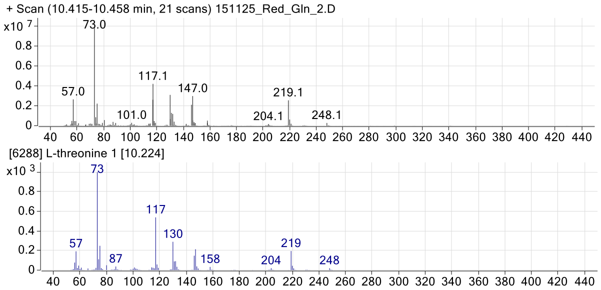

L-Glycine

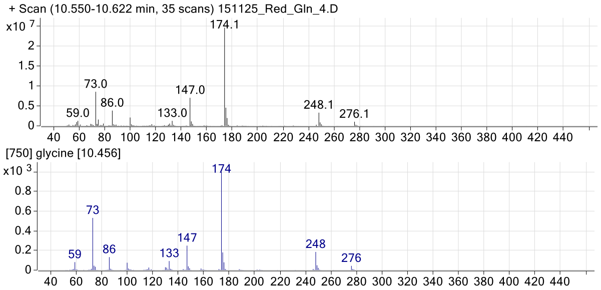

Succinic acid

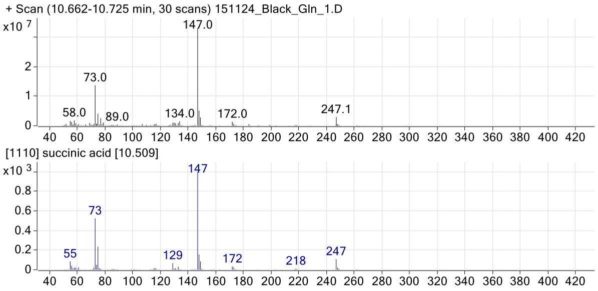

Fumaric acid

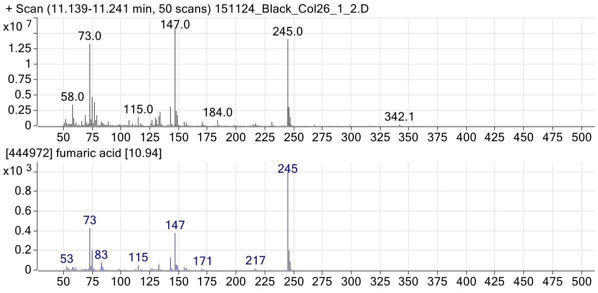

L-Homoserine

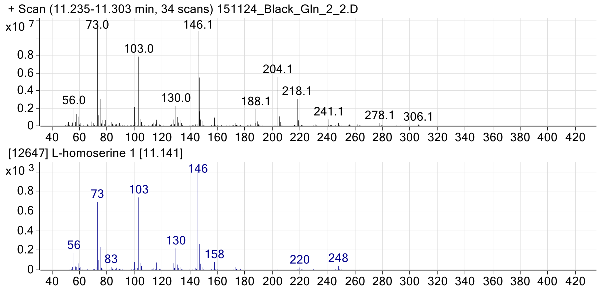

L-Alanine

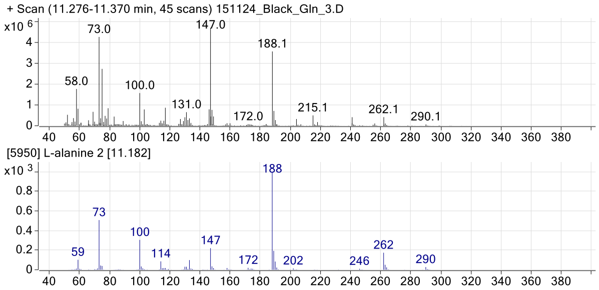

L-Methionine

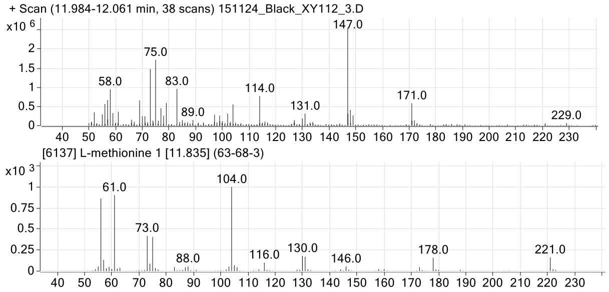

D-Malic acid

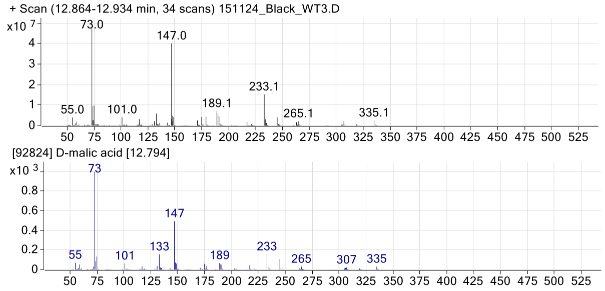

L-Aspartic acid

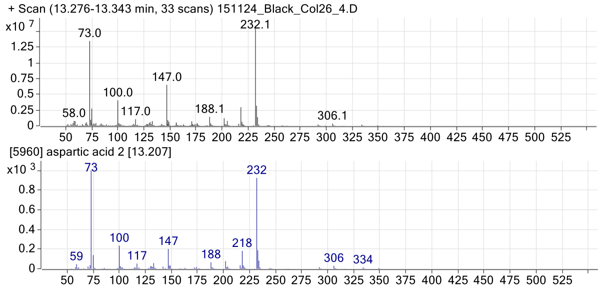

L-Glutamic acid

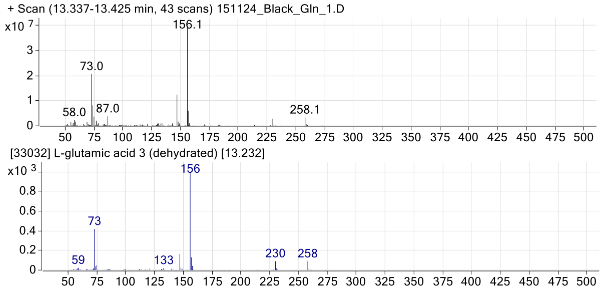

GABA

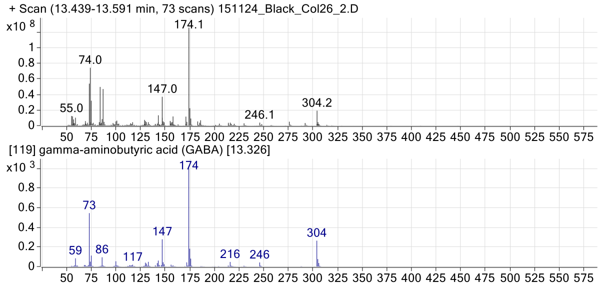

L-Cysteine

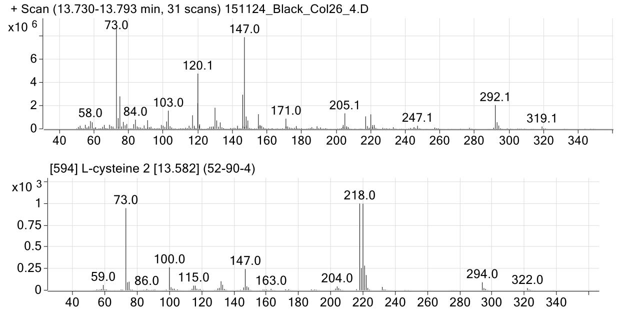

L-Phenylalanine

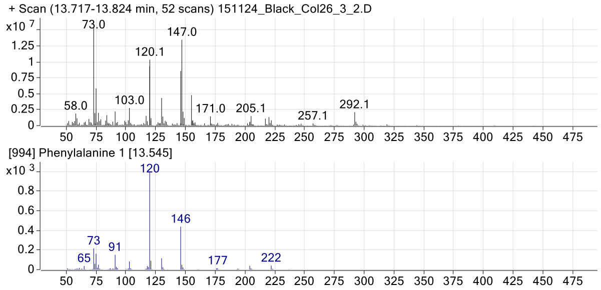

α-ketoglutarate

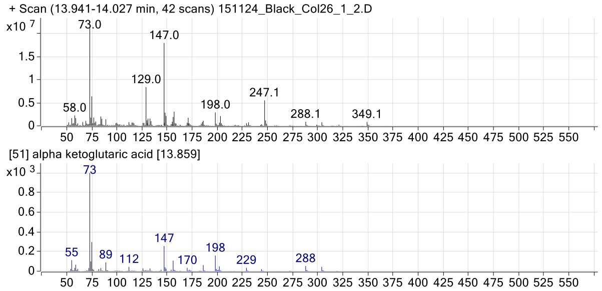

Phosphoenolpyruvic acid

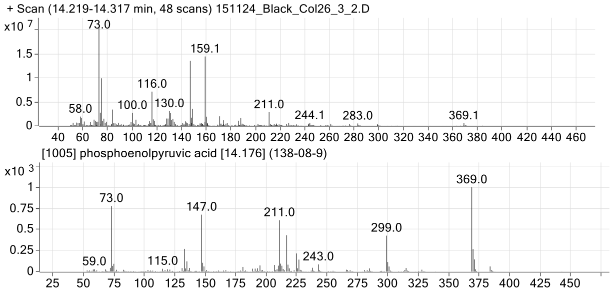

Putrescine

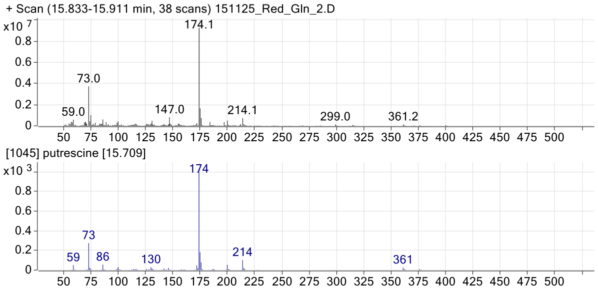

L-Glutamine

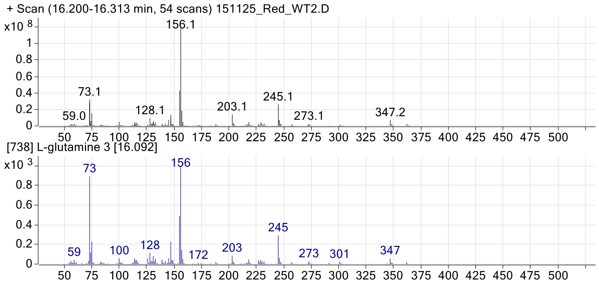

Citric acid

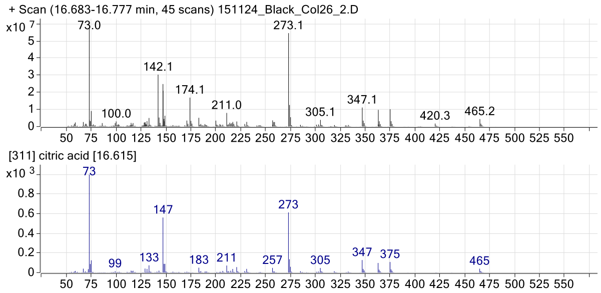

L-ornithine

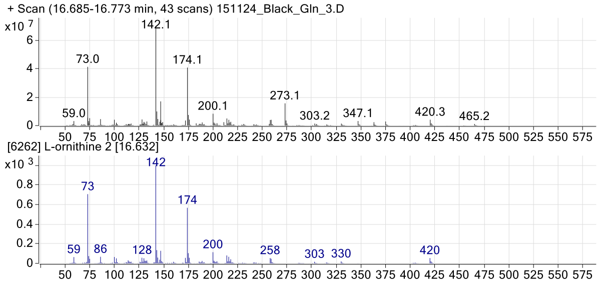

Citrulline

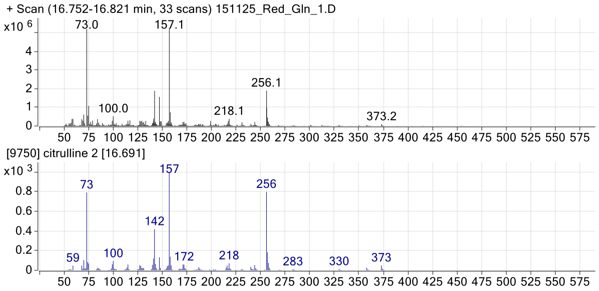

D-glucose

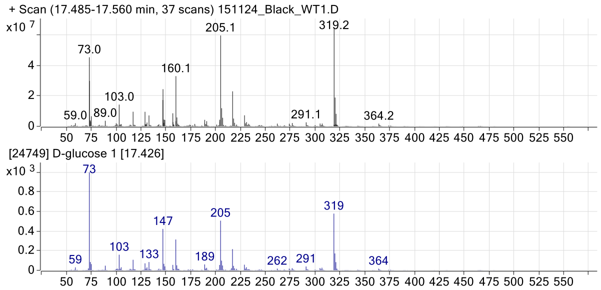

L-Histidine

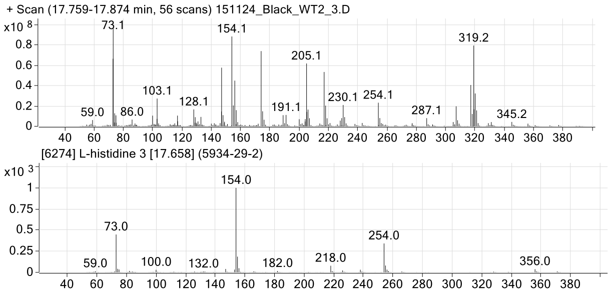

L-Lysine

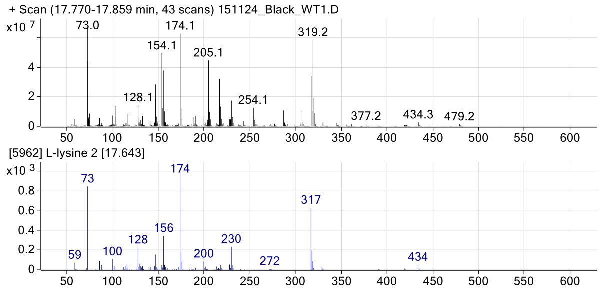

L-Tyrosine

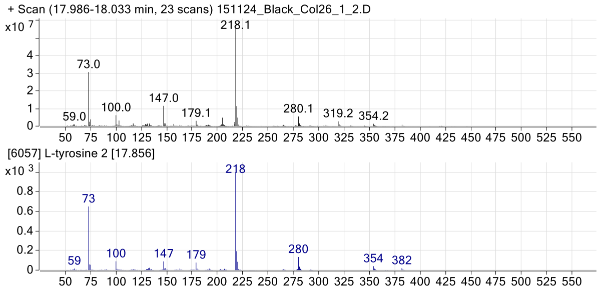

Palmitic acid

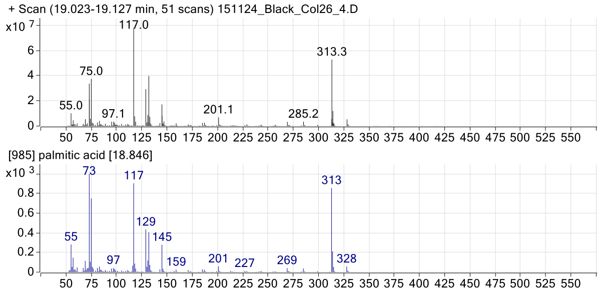

Allantoin

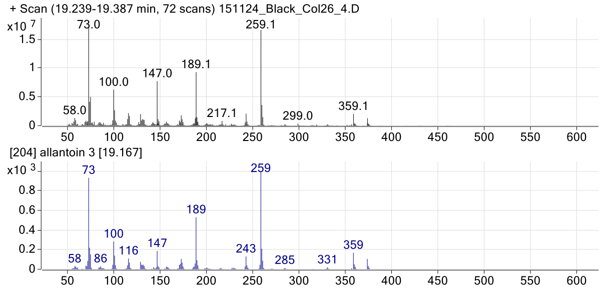

Uric acid

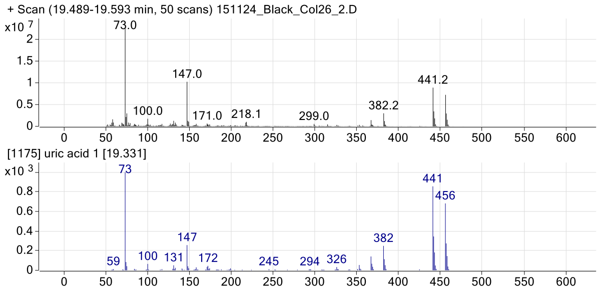

L-Tryptophan

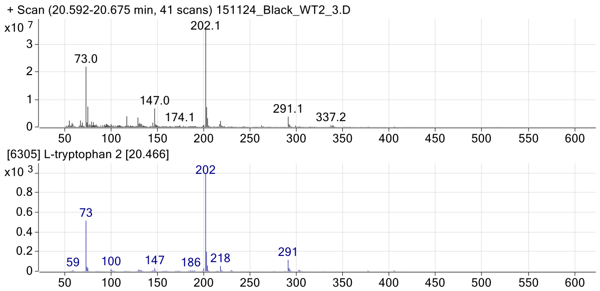

Linoleic acid

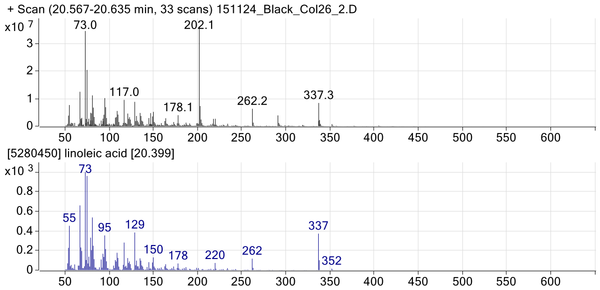

Oleic acid

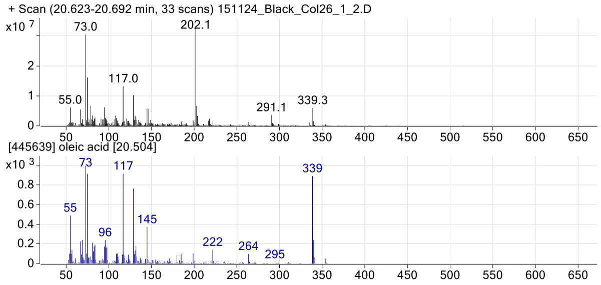

Stearic acid

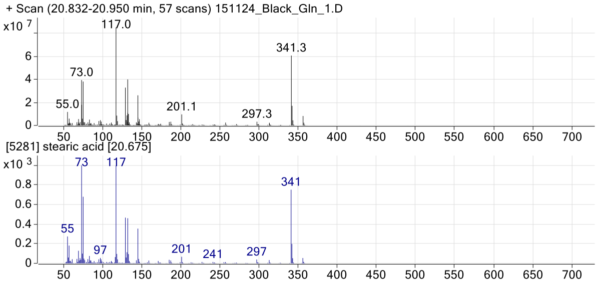

Fructose-6-phosphate

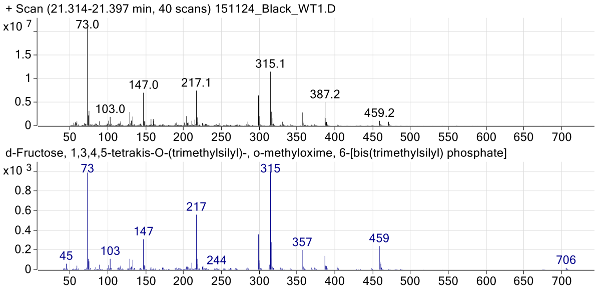

Glucose-6-phosphate

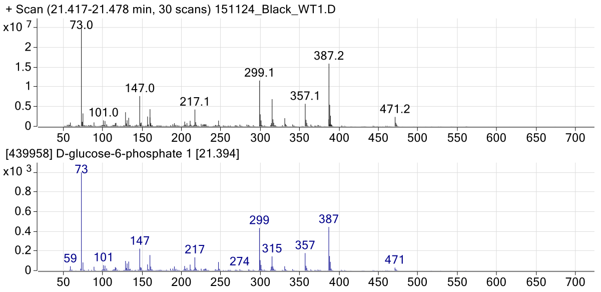

Trehalose

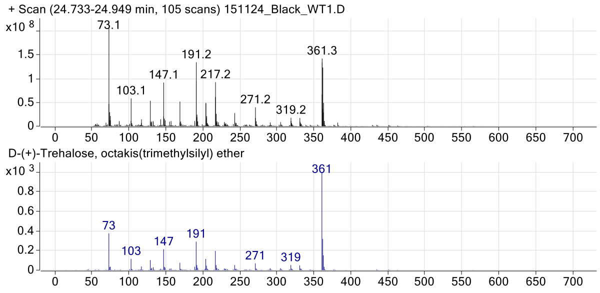

Squalene

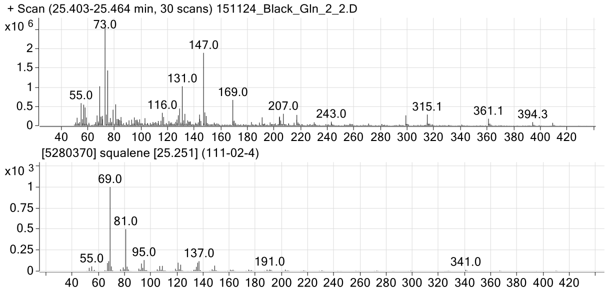

Ergosterol

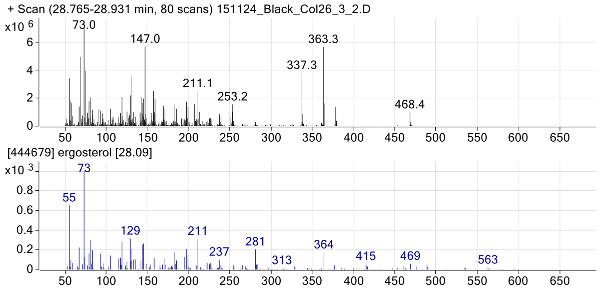

Supplement: S6 Table — (PDF) [file pgen.1006737.s006.pdf]
