# Supplementary material for: A fungal transcription factor essential for starch degradation affects integration of carbon and nitrogen metabolism
Source: PLoS Genet. 2017 May 3;13(5):e1006737. doi: 10.1371/journal.pgen.1006737 (PMC5435353; doi:10.1371/journal.pgen.1006737)
Supplement: S7 Table — (PDF) [file pgen.1006737.s007.pdf]

Table S7. Metabolites identified by LC-MS and corresponding ions used to extract peaks from raw data for relative quantitation.

Metabolite	Polarity	Formula	RT standard (min)	RT (min)	m/z [M-H]-	Identification
UDP-Glucose	Negative	C15H24N2O17P2	n/a	16.88	565.0477	MS/MS
Succinate semialdehyde	Negative	C4H6O3	4.91	4.98	101.0244	Standard
Pyruvate	Negative	C3H4O3	6.79	6.77	87.0088	Standard

UDP-Glucose

Ions in MS/MS mass fragmentation spectra of UDP-glucose in METLIN database, compared to MS/MS fragemtation spectra of observed peak in a representative sample.

Collision energy (V)	METLIN database MS/MS spectra major ions (%intensity)	Observed MS/MS spectra major ions (%intensity)
10	323.0270 (28)	323.0293 (30)
20	384.9852 (7), 323.0277 (100), 241.0115 (8), 78.9587 (9)	384.9825 (10), 323.0282 (100), 241.0096 (25), 78.9592 (28)
40	384.9835 (7), 323.0277 (37), 272.9565 (15), 241.0115 (18), 210.9990 (11), 158.9242 (19), 138.9778 (6), 111.0201 (11), 96.9692 (57), 78.9591 (100)	384.9823 (3), 323.0264 (15), 272.9565 (18), 241.0123 (15), 211.0041 (16), 158.9284 (12), 138.9789 (3), 111.0203 (16), 96.9697 (43), 78.9589 (100)

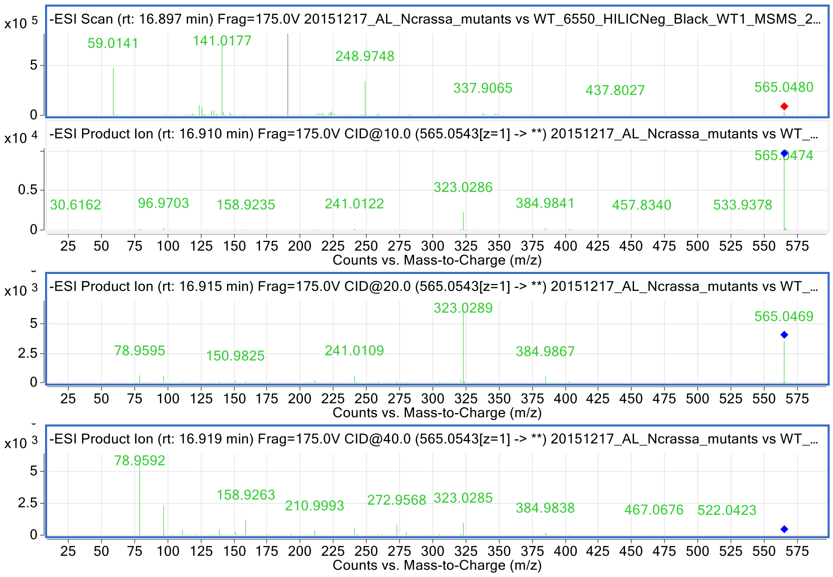

Mass spectra authentic reference standards compared to mass spectrum in representative sample

Succinate semialdehyde

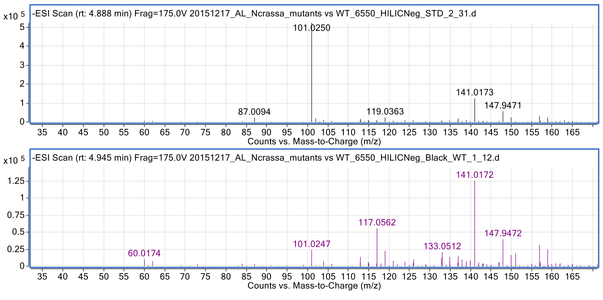

Pyruvate

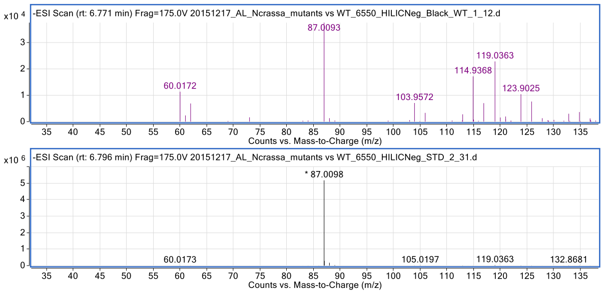
-ESI Scan (rt: 6.771 min) Frag=175.0V 20151217_AL_Ncrassa_mutants vs WT_6550_HILICNeg_Black_WT_1_12.d
x10 4
87.0093
119.0363
114.9368
123.9025
103.9572
60.0172
Counts vs. Mass-to-Charge (m/z)
-ESI Scan (rt: 6.796 min) Frag=175.0V 20151217_AL_Ncrassa_mutants vs WT_6550_HILICNeg_STD_2_31.d
x10 6
* 87.0098
60.0173
105.0197
119.0363
132.8681
Counts vs. Mass-to-Charge (m/z)
