# Supplementary material for: A fungal transcription factor essential for starch degradation affects integration of carbon and nitrogen metabolism
Source: PLoS Genet. 2017 May 3;13(5):e1006737. doi: 10.1371/journal.pgen.1006737 (PMC5435353; doi:10.1371/journal.pgen.1006737)
Supplement: S1 Fig — (PDF) [file pgen.1006737.s008.pdf]

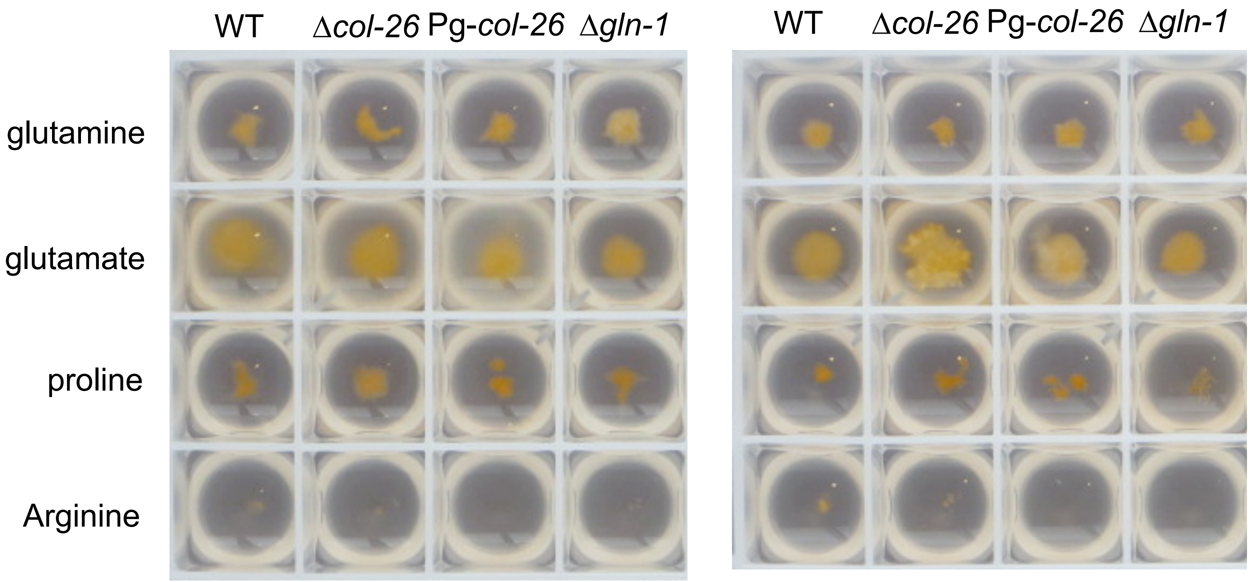

VMM with the amino acid as both carbon and nitrogen sources

VMM(NH_4NO_3) with the amino acid as the carbon source
