# Supplementary material for: A fungal transcription factor essential for starch degradation affects integration of carbon and nitrogen metabolism
Source: PLoS Genet. 2017 May 3;13(5):e1006737. doi: 10.1371/journal.pgen.1006737 (PMC5435353; doi:10.1371/journal.pgen.1006737)
Supplement: S2 Fig — (PDF) [file pgen.1006737.s009.pdf]

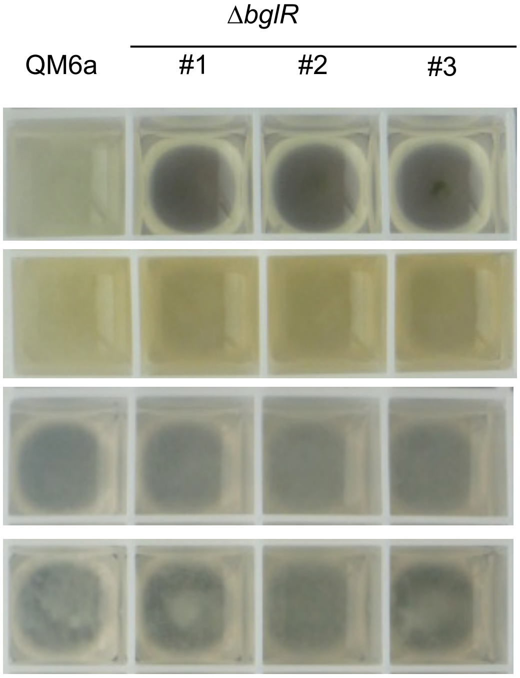

Carbon	Nitrogen	Time
glucose	ammonium sulfate	44 hours
glucose	glutamine	44 hours
glutamine	glutamine	48 hours
glutamine	ammonium sulfate	48 hours
